# Environment Changes Genetic Effects on Respiratory Conditions and Allergic Phenotypes

**DOI:** 10.1038/s41598-017-06791-y

**Published:** 2017-07-24

**Authors:** Yong Song, Michelle J. Schwager, Vibeke Backer, Jing Guo, Celeste Porsbjerg, Siew-Kim Khoo, Ingrid A. Laing, Eric K. Moses, Peter LeSouëf, Guicheng (Brad) Zhang

**Affiliations:** 1 School of Public Health, Curtin University, Kent St, Bentley, 6102 Western Australia, Australia; 2 Centre for Genetic Origins of Health and Disease, The University of Western Australia and Curtin University, 35 Stirling Highway, Crawley 6009 Western Australia, Australia; 3 School of Paediatrics and Child Health, The University of Western Australia, 35 Stirling Highway, Crawley 6009 Western Australia, Australia; 4 Department of Respiratory medicine, Bispebjerg University hospital, Copenhagen University, Copenhagen, Denmark; 5 Telethon Kids Institute, The University of Western Australia, 35 Stirling Highway, Crawley 6009 Western Australia, Australia

## Abstract

The prevalence of asthma and allergic diseases is disproportionately distributed among different populations, with an increasing trend observed in Western countries. Here we investigated how the environment affected genotype-phenotype association in a genetically homogeneous, but geographically separated population. We evaluated 18 single nucleotide polymorphisms (SNPs) corresponding to 8 genes (*ADAM33*, *ALOX5*, *LT-α, LTC4S*, *NOS1, ORMDL3, TBXA2R* and *TNF-α*), the lung function and five respiratory/allergic conditions (ever asthma, bronchitis, rhinitis, dermatitis and atopy) in two populations of Inuit residing either in the westernized environment of Denmark or in the rural area of Greenland. Our results showed that lung function was associated with genetic variants in *ORMDL3*, with polymorphisms having a significant interaction with place of residence. *LT-α* SNP rs909253 and rs1041981 were significantly associated with bronchitis risk. *LT-α* SNP rs2844484 was related to dermatitis susceptibility and was significantly influenced by the place of residence. The observed gene-phenotype relationships were exclusively present in one population and absent in the other population. We conclude that the genotype-phenotype associations relating to bronchitis and allergy susceptibility are dependent on the environment and that environmental factors/lifestyles modify genetic predisposition and change the genetic effects on diseases.

## Introduction

The global prevalence of asthma and other allergic conditions such as rhinitis, dermatitis and atopy, have been increasing for the past 30 to 40 years^1–3^. This rising trend is attributed to regions that are becoming more urbanised and ‘Westernised’^4^. The risk factors contributing to allergen hypersensitivity originate from an individual’s genetics, its accompanying lifestyles and the surrounding environment. To date, genetic association studies suffer from inconsistencies in identifying the susceptibility genes for asthma and allergy^5^. The genes/loci that are identified to be associated with the disease vary in different ethnic populations and in people from different geographic regions^5^. Thus, gene-environment interactions are likely to be critical determinants of asthma and allergy susceptibility.

The Inuit present a unique population to investigate the complex gene and environment interaction as they are homogenous and genetically distinct from other ethnicities^6^. This population descends from generations living in the harsh Artic environment. Their acculturation to the modern world, including migrating to Western countries, has brought a change in allergic condition prevalence in Inuit^7^. For the present study, we recruited two Inuit populations both born in Greenland but the first resided in their native country while the second migrated to the Western European country of Denmark. The comparison of the immigrants with Greenlandic Inuit is an ideal way to investigate how environmental factors affect genetic effects on disease susceptibility. The two Inuit populations were unselected for respiratory/allergic conditions, adjusted for several confounders particularly genetic admixture, and representative of the general Inuit population in each residing environment so that the findings resulting from this study can be generalised.

We selected 8 candidate genes which are related to asthma/allergic conditions or pulmonary function based on literature reports^8–14^. From these 8 genes, the genetic effects of 19 prospective single nucleotide polymorphisms (SNPs) on the lung function and five respiratory/allergic conditions (ever asthma, bronchitis, rhinitis, dermatitis and atopy) were compared in the two populations of Inuit. The interaction with place of residence was further investigated in the association studies. We hypothesised that the selected genetic variants were significantly associated with respiratory conditions or allergic prevalence in Inuit, and that the genetic effects were influenced by the residing environment or life styles. As differences of smoking condition and ancestral ethnicity were identified between the two populations in the current study, we also investigated the genotype-phenotype association profile, taking into account smoking effects and using the Inuit population with full ethnicity.

## Results

### Demographics

The demographic data for the two study populations are shown in Table 1. A lower proportion of Danish male Inuit (27.4%) compared to Greenlandic male Inuit (46.7%) was recruited in this study (*P* < 0.001). The proportion of smokers in the Greenlandic Inuit (88.1%) was higher than that in the Danish Inuit (78.8%, *P* < 0.001). In regards to the lung function, Greenlandic Inuit had lower values of FEV1 and FVC than Danish Inuit in both male and female populations (*P* < 0.05). After adjustment of gender, age, height, ethnicity and smoking status, this difference remained significant in the female population.

**Table 1:** Demographic and phenotypic data for Inuit residing in Greenland and Denmark.

		Greenland (n = 615)	Denmark (n = 643)	*P* value
Gender (male)		46.7 (287)	27.4 (176)	<0.001
Age (yr)		42.4 ± 14.78	43.4 ± 12.40	0.209
Height (cm)		163.1 ± 9.48	163.1 ± 10.10	0.995
Ethnicity^#^				
Full Inuit		91.5 (558)	53.2 (337)	
Non-full Inuit		8.5 (52)	46.8 (297)	<0.001
Smoker		88.1 (542)	78.8 (507)	<0.001
Lung Function				
FEV1 (L)	Male	3.7 ± 0.95	3.9 ± 0.89	0.017
	Female	2.7 ± 0.71	2.8 ± 0.64	0.016
FVC (L)	Male	4.7 ± 1.13	4.9 ± 1.04	0.041
	Female	3.3 ± 0.79	3.4 ± 0.72	0.014
Allergic/respiratory conditions				
Ever asthma		8.5 (52)	9.2 (59)	0.646
Bronchitis		25.6 (157)	13.9 (89)	<0.001
Rhinitis		42.7 (173)	60.0 (386)	<0.001
Dermatitis		57.7 (355)	36.3 (233)	<0.001
Atopy		24.0 (30)	23.4 (121)	0.896

Data are presented as Mean ± SD or % (n); ^#^Full Inuit means that all 4 grandparents are Inuit; Non-full Inuit means at least 1 non-Inuit grandparent.

**Figure 1:**
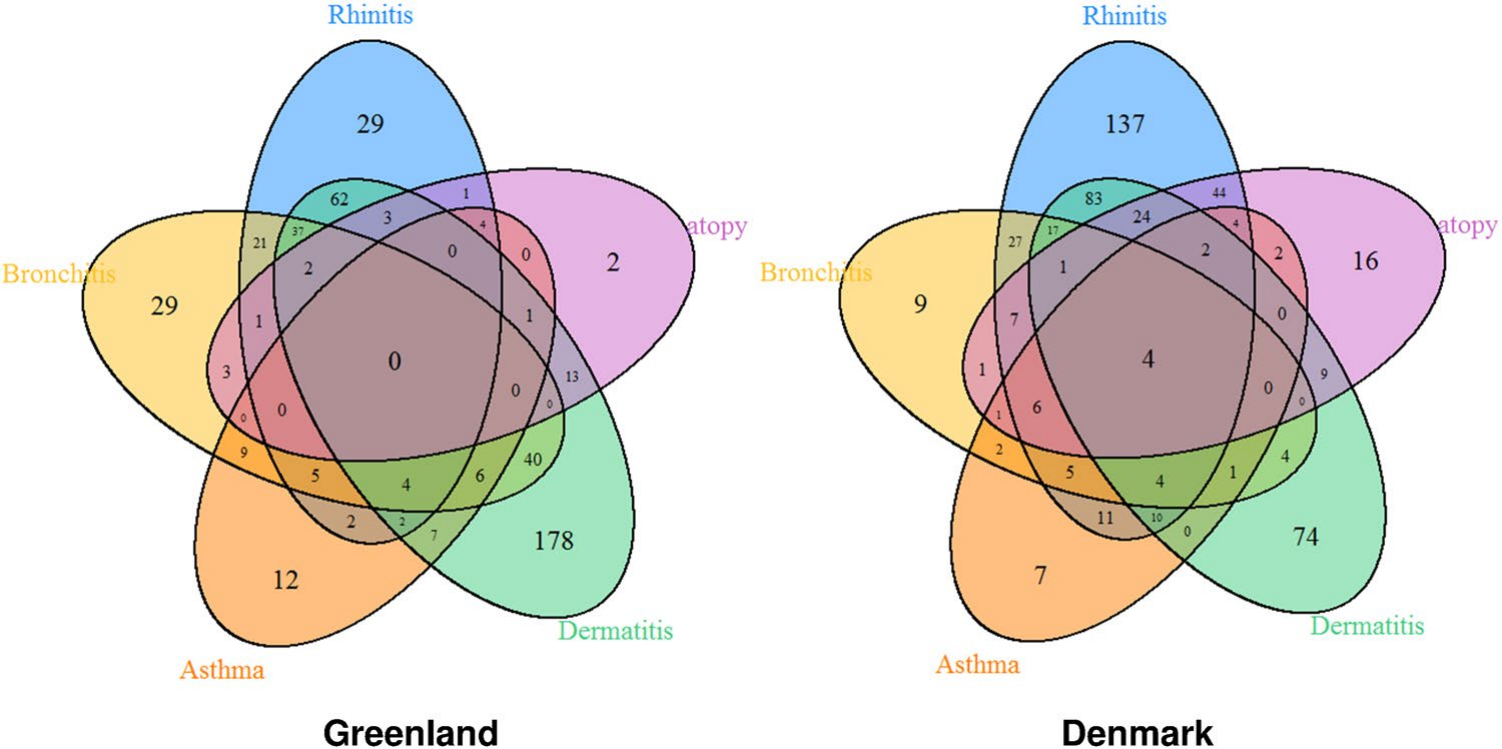
Interrelations of the respiratory/allergic diseases: Venn diagram shows the interrelations of the respiratory/allergic diseases in the two populations.

Inuit residing in Denmark had a higher prevalence of rhinitis (60.0% vs 42.7%, *P* < 0.001), but a lower prevalence of bronchitis (13.9% vs 25.6%, *P* < 0.001) and dermatitis (36.3% vs 57.7%, *P* < 0.001) compared with Greenlandic Inuit. These differences remained significant after adjustment for gender, age, height, ethnicity and smoking status. There was no significant difference between the two Inuit groups of ever asthma and atopy prevalence. Additionally these diseases were related to one another, as demonstrated by the interrelationship diagram in Fig. 1. We studied the relevance of different phenotypes by pooling the two Inuit populations together and adjusting for gender, age, height, ethnicity and place of residence and observed a positive correlation between smoking status and bronchitis prevalence and a negative correlation between lung function parameters (FEV1 and FVC) and bronchitis prevalence (*P* < 0.01).

### Genotype frequencies

We did not observe any deviation from the Hardy-Weinberg Equilibrium for all SNP genotype frequencies in both populations (*P* > 0.05; Supplementary Table S1). Apparent differences in genotype frequencies between the two Inuit populations (*P* < 0.05) were observed in *ADAM33* rs528557, the three *ALOX5* SNPs, *LT-α* rs2844484, *TBXA2R* rs4523, *TNF-α* rs1799964 and rs1800629. In the pure Inuit population (both grandparents and parents are Inuit), only significant differences were found for *ALOX5* SNPs (rs892690 and rs2115819), *TBXA2R* rs4523 and *TNF-α* rs1799964.

### Lung function parameters and SNPs

**Figure 2:**
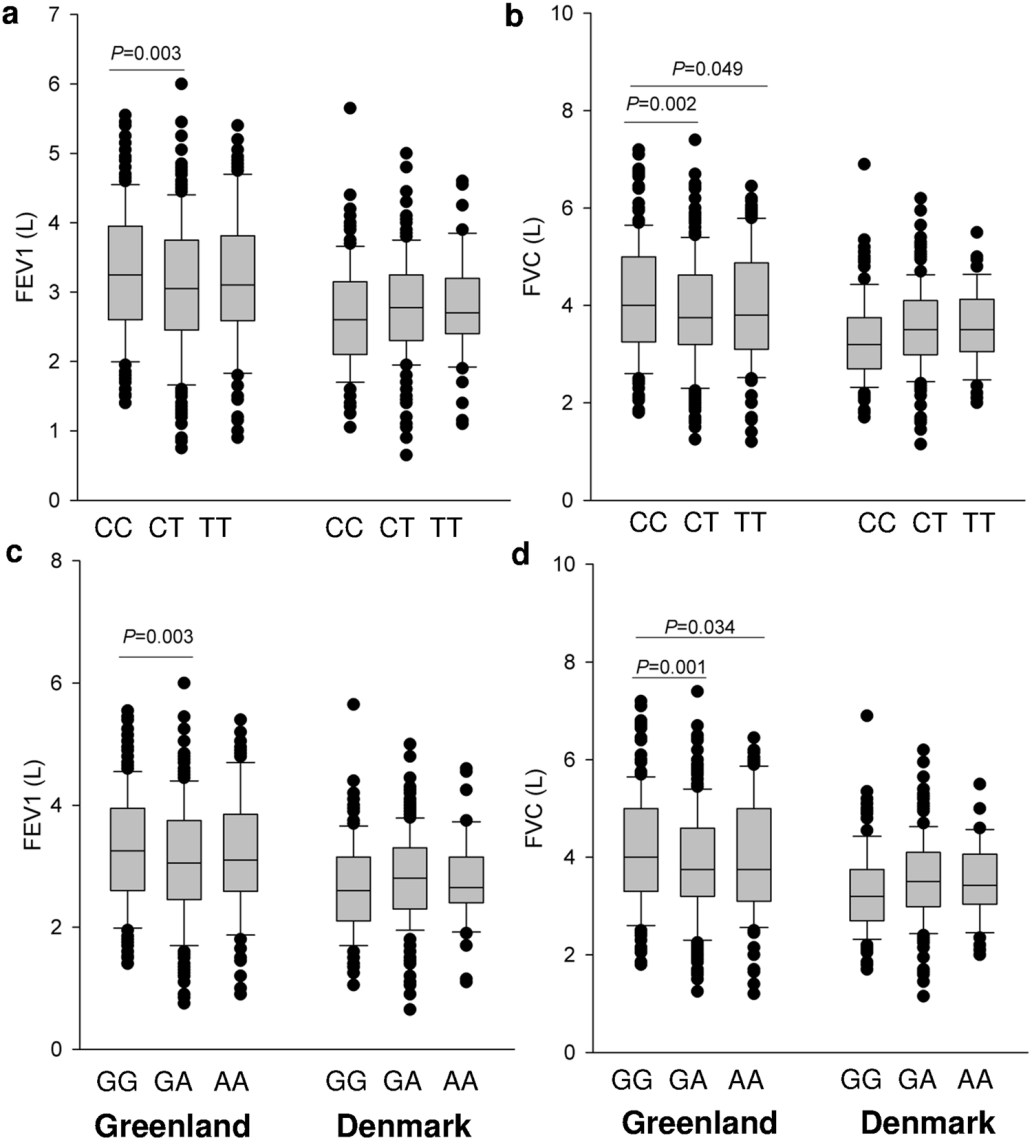
Associations of lung function and genotypes in the sensitivity analysis: Lung function (FEV1 and FVC) values in Greenlandic and Danish Inuit with full ethnicity were stratified by the genotypes of *ORMDL3* SNPs rs12603332 (**a** and **b**) and rs4065275 (**c** and **d**). *P* values were adjusted for age, gender, height, and smoking. Box and whisker plots represent median with 10th and 90th centiles.

The lung function data for the 18 SNP genotypes are shown in the Supplementary Table S2. After adjusting for gender, age, height, ethnicity and smoking status, *ORMDL3* SNPs (rs12603332 and rs4065275) were significantly associated with lung function (FEV1 and FVC) in Greenlandic Inuit whereas *ADAM33* rs2787094 was found to associate with FEV1 in Danish Inuit (*P* = 0.045). When we further tested these associations using the sensitivity analysis, the association of polymorphisms with lung function remained significant for *ORMDL3* SNPs (*P* < 0.05, Supplementary Table S8A), but not for *ADAM33* rs2787094 (*P* = 0.473, Supplementary Table S8B). The homozygous CC genotype of rs12603332 and the GG genotype of rs4065275 in *ORMDL3* were associated with higher values of FEV1 and FVC than the homozygous CT and GA genotypes in Greenlandic Inuit respectively (Fig. 2). Moreover, the interaction of the *ORMDL3* SNPs (rs12603332 and rs4065275) with place of residence were statistically significant for the lung function (FEV1 and FVC) (*P* < 0.05, Supplementary Table S9). Apart from these, we did not observe significant associations of any other SNPs with lung function in either the whole population or the full Inuit population.

### Ever asthma and SNPs

No significant difference in the prevalence of ever asthma was found for genotypes of the examined SNPs (Supplementary Table S3). However, in the sensitivity analysis, Greenlander with homozygous AA genotype of *ALOX5* SNP rs892690 had a significantly increased risk of ever asthma compared to GG genotype (*P* < 0.05, Supplementary Table S8A).

### Bronchitis and SNPs

Significant differences in the prevalence and risk of bronchitis were found in genotypes of *LT-α* and *TNF-α*, but not in *ALOX5*, *LTC4S*, *TBXA2R, ADAM33, NOS1* and *ORMDL3* in the Inuit populations residing both in Greenland and in Denmark (Supplementary Table S4). *LT-α* SNP rs2844484 was significantly associated with bronchitis, but only within the Danish population. On the other hand, two *TNF-α* SNPs (rs1800630 and rs1800629) were exclusively associated with bronchitis within the Greenlandic population. Using the sensitivity analysis, we further revealed the significant associations of *LT-α* rs909253 and rs1041981 with bronchitis in Danish Inuit, while the association of *TNF-α* rs1800630 with bronchitis did not remain significant (Supplementary Table S8).

**Figure 3:**
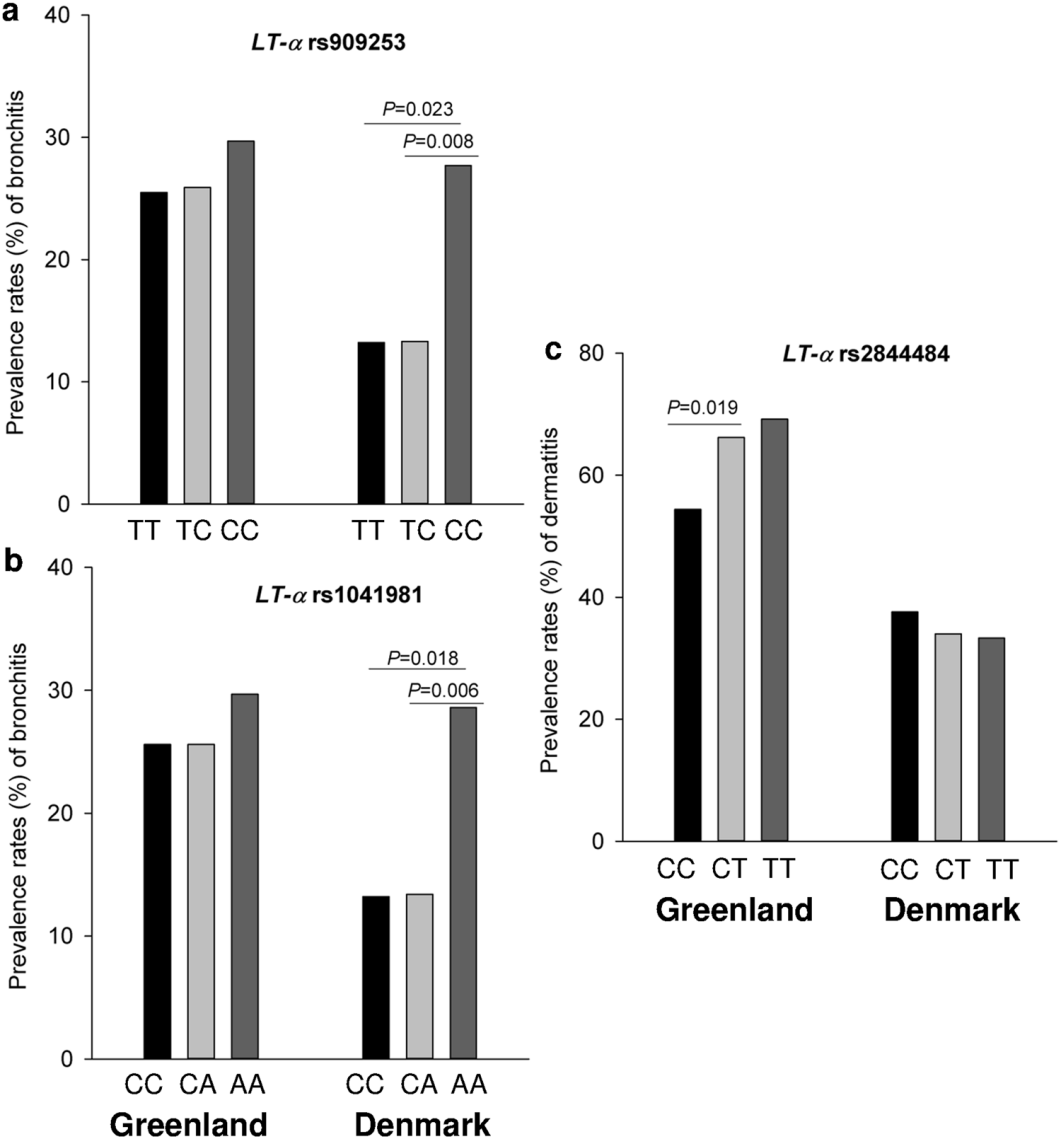
Prevalence of bronchitis/dermatitis and genotypes in the sensitivity analysis: Prevalence rates (%) of bronchitis in Greenlandic and Danish Inuit with full ethnicity were stratified by the genotypes of *LT-α* SNP rs909253 (**a**) and rs1041981 (**b**), while dermatitis prevalence was stratified by the genotypes of *LT-α* SNP rs2844484 (**c**). *P* values were adjusted for age, gender, height, and smoking.

The sensitivity analysis showed that in Danish Inuit, the prevalence of bronchitis was significantly lower among heterozygous CT for *LT-α* rs2844484 (8.8%) than among homozygous CC (19.4%) and TT (22.2%). Danish Inuit with genotype CC for *LT-α* rs909253 (Fig. 3a) and AA for and rs1041981 (Fig. 3b) had a significantly increased prevalence of bronchitis, compared to other genotypes. In Greenland, the Inuit with genotype GA of *TNF-α* rs1800629 had a significantly higher prevalence of bronchitis (38.9%) than that in GG (25.4%). However, interactions of the SNPs with place of residence were not statistically significant in the prevalence of bronchitis (Supplementary Table S9).

### Rhinitis and SNPs

No significant associations were found between rhinitis and any SNPs for Inuit residing in either Greenland or Denmark (Supplementary Table S5). Furthermore, the sensitivity analysis did not reveal any significant associations in the two populations (Supplementary Table S8).

### Dermatitis and SNPs

As shown in Supplemental Table 6, within the Greenlandic population, Inuit with *LT-α* rs2844484 homozygous CC (54.5%) had a significantly lower prevalence of dermatitis compared with Inuit with CT genotype (*P* = 0.035). The same significant relationship (*P* = 0.025, Fig. 3c and Supplementary Table S8A) was detected within the Greenlandic group using the sensitivity analysis. The place of residence variable was significantly interacted with *LT-α* rs2844484 on dermatitis (*P* = 0.009, Supplementary Table S9). No significant differences in the prevalence and risk of dermatitis and interaction with place of residence were found in other SNPs examined within the two Inuit populations.

### Atopy and SNPs

Significant differences in the prevalence and risk of atopy and association with atopy were only found in genotypes of *ALOX5* and *LTC4S* (Supplementary Table S7). However, these differences did not persist after the sensitivity analysis (Supplementary Table S8). There were no significant interactions for place of residence association with the SNPs on atopy (Supplementary Table S9).

### Correction for multiple tests

As shown in Table 2, the identified associations were further adjusted for multiple tests. The effects of *ALOX5* rs892690 on ever asthma, *LT-α* rs2844484 and *TNF-α* rs1800629 on bronchitis were not considered significant with the adjusted thresholds, while the others remained significant after the correction.

**Table 2:** Correction for multiple comparisons in the significant associations identified.

	Whole population (*P* value)	Full Inuit (*P* value)	*M*_*eff*_	Significance Threshold
*ORMDL3* rs12603332 & FEV1 in Greenland	0.058	0.014	1.0018	0.0253
*ORMDL3* rs12603332 & FVC in Greenland	0.027	0.009	1.0018	0.0253
*ORMDL3* rs4065275 & FEV1 in Greenland	0.033	0.011	1.0018	0.0253
*ORMDL3* rs4065275 & FVC in Greenland	0.023	0.005	1.0018	0.0253
*ALOX5* rs892690 & Ever asthma in Greenland	0.025	0.029	2.686	0.0170
*LT-α* rs2844484 & Bronchitis in Denmark	0.002	0.044	1.8635	0.0253
*LT-α* rs909253 & Bronchitis in Denmark	0.090	0.019	1.8635	0.0253
*LT-α* rs1041981 & Bronchitis in Denmark	0.070	0.014	1.8635	0.0253
*TNF-α* rs1800629 & Bronchitis in Greenland	0.035	0.023	2.7127	0.0170
*LT-α* rs2844484 & Dermatitis in Greenland	0.035	0.025	1.8635	0.0253

*M*_*eff*_ : the effective number of SNPs tested within each gene.

### Genotype and gene expression associations

Associations between genotype of the selected SNPs (*ORMDL3* rs12603332 and rs4065275, *LT-α* rs909253, rs1041981 and rs2844484) and blood-specific gene expression levels were analysed using GTEx portal. We revealed that *ORMDL3* gene expression was up-regulated in blood cells of people with allele T of rs12603332 (*P* = 1.1e-25) or allele A of rs4065275 (*P* = 1.6e-25). The polymorphisms of *LT-α* rs909253 and rs1041981 were not significantly related to mRNA expression of *LT-α* (*P* > 0.05). Compared to CC, genotype CT of rs2844484 was related to a higher *LT-α* gene expression (*P* = 0.0065).

## Discussion

In this study, we compared the genetic effects of 18 single nucleotide polymorphisms in 8 asthma and allergic candidate genes on the lung function and respiratory/allergic conditions in two populations of Inuit: one born and residing in Greenland and the other first generation Inuit immigrants of Denmark. We discovered several significant associations with respiratory/allergic conditions in the eight candidate genes after adjusting for the confounding factors (e.g. gender, age, height, ethnicity and smoking), using the sensitivity analysis and correcting for multiple testing. These include 1) Lung function parameters are associated with genetic variants in *ORMDL3*, with polymorphisms having a significant interaction with place of residence; 2) Frequency of bronchitis is associated with variants in the *LT-α* gene; 3) Dermatitis risk is related to the genotype distribution of *LT-α* SNP rs2844484, with a significant interaction with place of residence. The gene-phenotype relationship was exclusively observed in one population and not the other, indicating that environment plays a vital role of regulating the genetic effects.

Comparison of the pulmonary function in the two populations indicated that Denmark Inuit had higher lung function FEV1 and FVC values than Greenlandic Inuit. However, after correcting for the confounding variables (e.g. gender, age, height, ethnicity and smoking status) in the analysis, we found that only female Inuit in Denmark had a better lung function performance than the corresponding population in Greenland. In analysing the respiratory conditions and allergic phenotypes, we did not observe a significant difference of prevalence in asthma, but a higher prevalence of rhinitis and a lower prevalence of bronchitis (13.9%) and dermatitis (36.3%) in Danish Inuit compared with Greenlandic Inuit. The high prevalence of bronchitis and dermatitis in Greenlandic Inuit observed in this study is consistent with the reported frequency of these diseases in Greenlander^7^. Indigenous Inuit were previously reported to have reduced respiratory health due to high tobacco consumption^7^. In the present study, we also noted that the proportion of smokers was higher in Greenlandic Inuit compared to Danish Inuit. A positive association between bronchitis and smoking status and a negative association between bronchitis frequency and lung function were revealed when investigating two populations as a whole. Thus, we conclude that smoking tobacco is a risk factor for bronchitis, and the development of bronchitis is likely to contribute to the compromised lung function of Inuit.

Out of the 18 SNPs that we examined, *ORMDL3* variants (rs12603332 and rs4065275) were significantly associated with lung function in Greenlandic Inuit after controlling the confounding variables and showed an interaction with place of residence. ORMDL3 is involved in endoplasmic reticulum–mediated Ca^2+^ homeostasis and unfolded protein response which potentially activate inflammatory processes and T-lymphocyte induction^15^. We found that the T allele of rs12603332 and the A allele of rs4065275 had a reduced lung function compared to the C or G allele, respectively. A change from C to T allele in rs12603332 was reported to result in an increase in *ORMDL3* transcription^16^. Indeed, according to the GTEx database^17^, *ORMDL3* rs12603332 and rs4065275 are expression quantitative trait loci (eQTL). Allele T of rs12603332 or allele A of rs4065275 was correlated to overexpression of *ORMDL3* gene. Thus, increased ORMDL3 may provoke an inflammatory response in lung tissue and affect pulmonary performance. It should be noted that *ORMDL3* is well known as an asthma susceptibility gene as confirmed in genetic association studies in several ethnic populations^16^ and also associated with smoking exposure^18^. Further testing on how smoking and other environmental factors regulate the effect of *ORMDL3* SNPs on lung function is of interest, particularly in light of the uncertainty surrounding ORMDL3’s direct relation to asthma pathogenesis.

We also found that Danish Inuit with the minor allele homozygous genotypes CC and AA for rs909253 and rs1041981 in *LT-α*, respectively, had 2-fold increased risk of developing bronchitis. *LT-α* rs909253 is located in the first intron of the gene while *LT-α* rs1041981 is located in the chromosomal region that transcribes for codon 60 and translates into an amino acid change, from threonine to asparagine. The C allele of rs909253 was reportedly associated with increased synthesis of LT-α^19^. However, GTEx analysis did not support that the polymorphisms of both variants were significantly related to functional expression of *LT-α*. Interestingly, these SNPs were confirmed to be in high linkage disequilibrium by a genome wide association study on lung function^20^ and the current study (Supplementary Fig. 1). Individuals with a haplotype consisting of CC genotype of rs909253 and AA genotype of rs1041981 produced substantially higher amounts of LT-α in their peripheral blood mononuclear cells^21^. Thus, the individual SNPs may have minimal functional impact and it is likely that *LT-α* rs909253 and rs1041981 affect the functional expression in linkage disequilibrium forming a functional haplotype. Functionally, LT-α is a major player of the lymphotoxin signalling pathway in regulating the development and maintenance of secondary lymphoid organs and controlling organ design^22^. Previously it was reported that rs909253 was associated with pulmonary function in a cohort of Caucasian smokers^23^. Since the inflammation response to external environmental factors involves this cytokine, the bronchitis associations found in Inuit further support that *LT-α* play a biological or pathological role in the development of bronchitis in the Inuit population. In addition, the two genetic variants in *LT-α* were only associated with bronchitis in Danish Inuit, but not in Greenlandic Inuit. These findings indicate that the environmental factors interact with different genetic variants and alter the genetic effects on bronchitis. However, living place was not significantly interacted with the genotype–phenotype associations. Other important factors were proposed in the development of respiratory illness in Greenlandic Inuit, including indoor chimney heating, dry frost at low temperatures, passive smoke exposure during infancy, respiratory infections during childhood etc^7^. Studies are needed to explore whether these environmental factors are responsible for mediating genetic predisposition of *LT-α* to bronchitis.

A genetic variant (rs2844484) in the *LT-α* gene was also revealed to associate with dermatitis. *LT-α* rs2844484 is positioned upstream from the start of *LT-α* and this SNP is part of a region identified as a hypersensitive site shown to contain upstream stimulatory factors^24^. This family of transcription factors has the capability to vary *LT-α* expression when under stress and immune-related conditions^24^. It is also suggested that LT-α plays a crucial role in the inflammation mechanism associated with immune responses to infectious agents, by stimulating chemokines and adhesion molecules^25^. When infectious agents including foreign antigens and pathogens invade the skin, ectopic lymphoid tissue develops under the regulation of LT-α^22^. We found that Greenlandic Inuit with minor allele (CT) in the *LT-α* SNP rs2844484 had an increased risk of dermatitis, and the place of residence is significantly interacted with the association of *LT-α* SNP rs2844484 with dermatitis prevalence. In addition, the GTEx data also supported that individual with genotype CT of rs2844484 had a higher *LT-α* gene expression, relative to CC. Considering the regulatory role of LT-α cytokine in the immune defensive system, our data suggest that the *LT-α* gene variant affects the dermatitis prevalence, and this association is regulated by environmental factors.

One limitation of our study is that the sample size is small after stratifying by genotype, particularly for SNP genotypes associated with low disease prevalence. Nevertheless, we identified several significant genetic effects as well as interactions with place of residence after testing in the sensitivity analysis and correcting for multiple tests. The relatively small sample size for the population study also reduces the statistical power and has potentially type 2 errors (false negative). A larger sample size population with a similar genetic background is needed to validate or replicate our results. Consequently, the results should be interpreted with caution and we encourage further studies to confirm these findings.

Taken together, our data show that polymorphisms of *ORMDL3* are related to lung function and affected by place of residence. Moreover, the candidate gene *LT-α* variants are significantly associated with either respiratory inflammation or allergic conditions. The effects of heterozygote on respiratory function and allergic traits are different in these associations. We argue that the protective effect of heterozygote is partly attributed to the natural consequence of adaptation, in which the fitness of the heterozygote is superior to either homozygote^26^. The associations of the heterozygous genotypes with lung function and disease prevalence may also result from potential linkage of the SNP with other functional genetic variants or by a chance. The results should be interpreted with caution. Interestingly, these associations are environment dependent, indicating that environmental factors or life styles could modify genetic predisposition, and change the genetic effects on diseases.

## Materials and Methods

### Study populations

Two populations of Inuit were studied, the Inuit population residing in Qasigiannguit (North/West Greenland) and the first generation Inuit immigrants residing in Denmark. The mean length of residence in Denmark of the migrants was 23 years and they had lived 52% of their life in Denmark. The two Inuit populations were originally recruited as part of a larger study conducted by The Greenlandic Study Population Group^27^. All Inuit from both populations were born in Greenland. Inuit ethnicity was self-reported and ethnicity was documented by number of Inuit grandparents for each subject^27^.

Qasigiannguit is a small town, near the Arctic Circle, which is only accessible by boat during the short summer season. A high proportion of this population works outside as fishermen or hunters (60.4%) and consumes mainly traditional Greenlandic food (84.9%), such as marine mammals^7^.

The second Inuit population examined in this study are first generation immigrants residing in Copenhagen, the capital of Denmark. Copenhagen residents are considered to live in a predominantly ‘Western’ European culture and lifestyle. A low proportion of first generation immigrant Inuit in Denmark works as fishermen or hunters (0.4%), and rarely consumes traditional Greenlandic food (4.5%)^7^.

DNA was available of 625 Inuit from Qasigiannguit and 680 Inuit from Denmark of which 98.4% (*n* = 615) and 94.6% (*n* = 643) provided phenotypic data, respectively. The genotype and phenotype study was approved by the ethics committees of Bispebjerg University hospital, Denmark, and School of Paediatrics and Child Health, the University of Western Australia. All subjects gave informed consent prior to participation. All experiments were performed in accordance with relevant guidelines and regulations of Bispebjerg University hospital and School of Paediatrics and Child Health, the University of Western Australia.

### Study design

This study investigated the environmental interaction between the individual genetic effects of the eight candidate genes (*ADAM33*, *ALOX5, LT-α*, *LTC4S*, *NOS1*, *ORMDL3*, *TBXA2R*, and *TNF-α*) on lung function and allergic conditions in Greenlandic and Danish Inuit. The cohorts are an unselected sample of adult participants, representative of their respective Inuit populations. The original data collection occurred during the years 1997–2001^27^. This comprised of an interview, a questionnaire, clinical examinations, lung function studies and blood sample collection, and was performed by a team of seven investigators, consisting of trained staff from Denmark and staff from the local hospital with constant supervision by a senior investigator from the Greenlandic Population Study Group^27^.

### Phenotypic characterization

Respiratory and allergic conditions (ever asthma, bronchitis, rhinitis, dermatitis and atopy) were initially defined by the questionnaire^27^. Atopy was further determined using skin prick tests (SPTs) according to the European Academy of Allergy and Clinical Immunology (EAACI) guidelines. The allergens used for SPTs are commonly present in both Greenlandic and Danish environments, including birch, timothy grass, mugwort, animal dander (horse, dog, and cat), house dust mites (*Dermatophagoides pteronyssinus* and *D. farina*) and moulds (*Alternaria alternate* and *Cladosporium herbarum*). Atopy was recorded if a wheal size >3 mm to one of the allergens^27^.

Lung function parameter values were calculated from the completed capacities using a 7L wedge spirometer (Vitalograph Spiropharma, Copenhagen, Denmark), including reduced expiratory volume in one second (FEV1) and forced vital capacity (FVC) values^28^.

### Selection of single nucleotide polymorphism (SNP)

**Figure 4:**
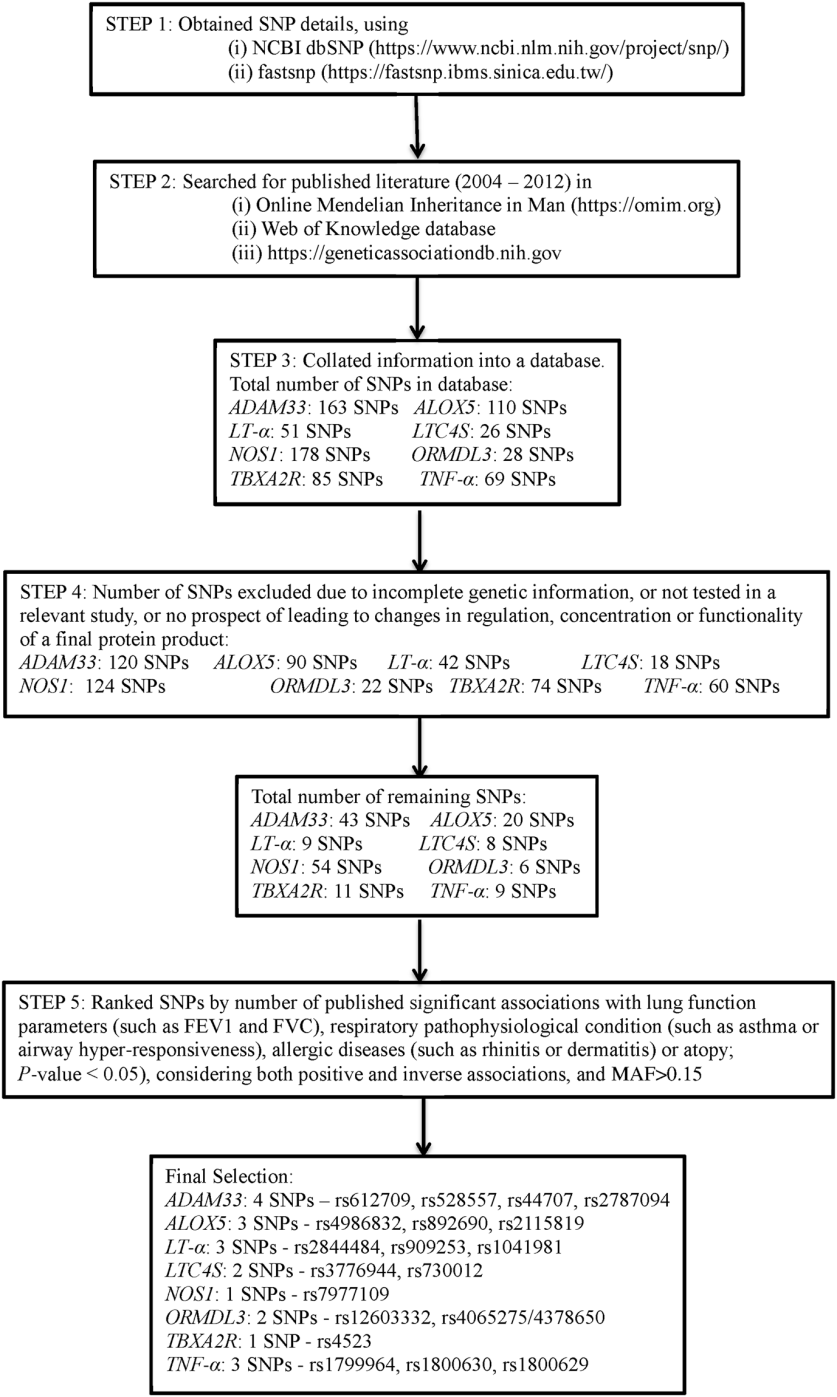
Selection of single nucleotide polymorphisms (SNPs): Flow chart summarises the procedures for the selection of SNPs in the study. MAF: minor allele frequency.

The SNP selection criteria are summarized in the flowchart of Fig. 4. Specifically, the search started with the Online Mendelian Inheritance in Man website (https://omim.org) to obtain historical molecular genetics information and landmark papers about each candidate gene. Subsequently the Web of Knowledge database was employed to retrieve possible candidate gene SNPs from literatures published from 2004 to 2012. The Genetic Association Database (https://geneticassociationdb.nih.gov) was aligned to obtain other possible references using the “broad phenotype (disease)” category. NCBI dbSNP databases (http://www.ncbi.nlm.nih.gov/SNP/) and fastsnp (https://fastsnp.ibms.sinica.edu.tw/) were used as additional resources for analysing SNP details. The SNP information was collated into a database, e.g. the rs number, minor allele frequency (MAF), location within gene, associations with asthma and allergic diseases, ethnicity. Finally, the recorded SNPs from each candidate gene were ranked by *P* value, irrespective of positive or negative associations. SNPs were selected based on significant association and with MAF > 0.15. We selected 19 SNPs for the study: *ADAM33* (rs612709, rs528557, rs44707 and rs2787094); *ALOX5* (rs4986832, rs892690 and rs2115819); *LT-α* (rs2844484, rs909253 and rs1041981); *LTC4S* (rs3776944 and rs730012); *NOS1* (rs7977109); *ORMDL3* (rs12603332 and rs4065275/4378650); *TBXA2R* (rs4523); *TNF-α* (rs1799964, rs1800630 and rs1800629);

### Genotyping

The genomic DNA was prepared from peripheral blood using standard methods^27^. The DNA was then genotyped for the selected SNPs using the Sequenom MassARRAY® system, with the iPLEX Gold primer extension process and MALDI-TOF mass spectrometry analysis. All SNPs were successfully genotyped except *LTC4S* SNP rs3776944 that was thereafter not included in the analysis.

### Genotype-tissue expression (GTEx) analysis

The five SNPs (*ORMDL3* rs12603332 and rs4065275, *LT-α* rs909253, rs1041981 and rs2844484) were finally selected for further analysis. Utilizing the GTEx database (http://gtexportal.org/home, version V6)^17^, we investigated the association between genotypes and gene expression level in whole blood.

### Statistical analysis

Assessments of Hardy Weinberg Equilibriums (HWE) were obtained via the Innate Immunity Programs for Genomic Applications collaboration website (https://regepi.bwh.harvard.edu/IIPGA2/Bioinformatics/). The genotype frequencies were compared using Pearson χ^2^ test, further stratified by ethnicity. All genotype-phenotype association analyses were performed using a co-dominant model in which genotypes of SNPs were treated as categorical variables. Specifically, the associations were investigated using linear regression between SNPs and lung function and using logistic regression analyses between SNPs and disease prevalence. The regression analyses were adjusted for potential confounders e.g. gender, age, height, ethnicity, and smoking. The outcome variable was either the value of FEV1 and FVC or the frequency of respiratory/allergic phenotypes (Yes/No), while the independent variables were SNP genotype and the potential confounders. A sensitivity analysis was further performed by analysing the genotype-phenotype associations in Inuit with full ethnicity (all four grandparents are Inuit).

The interactive effect of place of residence on genotype-phenotype associations was investigated by using the likelihood-ratio test after estimation in the regression models by pooling the two populations together. Both the unrestricted (with interaction) and the restricted (without interaction) models were fitted using the maximum-likelihood method, and subsequently these were compared to identify significant interactions. Differences with *P* < 0.05 were considered statistically significant.

All analyses mentioned above were conducted using the statistical package SPSS (version 20.0: SPSS Inc., Chicago, IL, USA) except for the likelihood-ratio tests for which STATA (StataCorp LP, College Station, TX, USA) was used.

In addition, a standard Bonferroni correction for multiple testing is considered too conservative without taking into account linkage disequilibrium between SNPs in the same gene. This can result in a significant overcorrection and subsequent loss of power. Inconsistencies that arise from population differences may be resolved by using a gene based approach rather than either a SNP-based or a haplotype-based approach^29, 30^. As the strong linkage disequilibrium was identified in the present study (Supplementary Fig. 1), we employed a gene-based approach and applied multiple comparison correction with respect to the number of SNPs in that gene. We used the method proposed by Li and Ji^31^ to calculate the number of independent components underlying the linkage disequilibrium structure of the set of SNPs, as implemented in Single Nucleotide Polymorphism Spectral Decomposition (SNPSpD)^32^.

### Data Availability

The datasets generated during and/or analysed during the current study are available from the corresponding author on reasonable request.

## Electronic supplementary material

Supplementary Information
