# Supplementary material for: Environment Changes Genetic Effects on Respiratory Conditions and Allergic Phenotypes
Source: Sci Rep. 2017 Jul 24;7:6342. doi: 10.1038/s41598-017-06791-y (PMC5524954; doi:10.1038/s41598-017-06791-y)
Supplement: Supplementary file 1 — Supplementary Information [file 41598_2017_6791_MOESM1_ESM.pdf]

Environment Changes Genetic Effects on Respiratory Conditions and Allergic Phenotypes

*Yong Song[1, 2], Michelle J. Schwager[3], Vibeke Backer[4], Jing Guo[1, 2], Celeste Porsbjerg[4], Siew-Kim Khoo[3], Ingrid A. Laing[3], Eric K. Moses[2], Peter LeSouëf[3], Guicheng (Brad) Zhang[1, 2, 3]**

[1] *School of Public Health, Curtin University, Kent St, Bentley, 6102, Western Australia, Australia*

[2] *Centre for Genetic Origins of Health and Disease, The University of Western Australia and Curtin University, 35 Stirling Highway, Crawley, 6009, Western Australia, Australia*

[3] *School of Paediatrics and Child Health, The University of Western Australia, 35 Stirling Highway, Crawley, 6009, Western Australia, Australia*

[4] *Department of Respiratory medicine, Bispebjerg University hospital, Copenhagen University, Denmark*

Yong Song and Michelle J Schwager contributed equally to the study.

*Address correspondence to:

Guicheng (Brad) Zhang (PhD); Address: School of Public Health, Curtin University of Technology, Kent St, Bentley, Western Australia 6102, Australia; Tel: +61 8 9266 3226; Fax: +61 8 9266 9266; Email: brad.zhang@curtin.edu.au

SUPPLEMENTARY TABLES

Supplementary Table S1: Genotype frequencies, minor allele frequencies and Hardy-Weinberg Equilibrium probabilities for the SNPs in Greenlandic and Danish Inuit populations.

	Greenland				Denmark				
	N	%	MAF	HWE	N	%	MAF	HWE	*P*
ADAM33									
**rs612709**									
GG	540	87.9	A: 6.1%	0.719	548	85.2	A: 7.8%	0.576	0.161
GA	73	11.9			90	14.0			
AA	1	0.2			5	0.8			
**rs528557**									
CC	493	81.0	G: 10.3%	0.126	464	72.5	G: 14.7%	0.639	**0.002**
CG	106	17.4			164	25.6			
GG	10	1.6			12	1.9			
**rs44707**									
AA	276	45.3	C: 32.6%	0.927	290	45.2	C: 31.7%	0.144	0.482
AC	269	44.2			295	46.0			
CC	64	10.5			56	8.8			
**rs2787094**									
GG	490	80.1	C: 10.4%	0.663	489	76.0	C: 12.8%	1.000	0.157
GC	117	19.1			144	22.4			
CC	5	0.8			10	1.6			
ALOX5									
**rs4986832**									
GG	542	88.9	A: 5.8%	0.449	524	81.5	A: 9.9%	0.382	**0.001**
GA	65	10.7			111	17.3			
AA	3	0.5			8	1.2			
**rs892690**									
GG	272	45.0	A: 32.1%	0.263	212	33.3	A: 42.1%	0.935	**<0.001**
GA	277	45.8			312	49.1			
AA	56	9.2			112	17.6			
**rs2115819**									
CC	135	22.2	C: 47.1%	1.000	190	29.7	T: 44.3%	0.200	**<0.001**
CT	304	49.9			332	52.0			
TT	170	27.9			117	18.3			
LT-α									
**rs2844484**									
CC	440	72.5	T: 15.0%	0.634	380	59.2	T: 22.5%	0.257	**<0.001**
CT	152	25.0			235	36.6			
TT	15	2.5			27	4.2			
**rs909253**									
TT	180	29.6	C: 44.4%	0.190	200	31.2	C: 43.0%	0.172	0.761
TC	317	52.0			332	51.7			
CC	112	18.4			110	17.1			
**rs1041981**									
CC	183	30.0	A: 44.2%	0.251	201	31.5	A: 42.6%	0.169	0.712
CA	316	51.7			330	51.7			
AA	112	18.3			107	16.8			
LTC4S									
rs730012									

	Greenland				Denmark				
	N	%	MAF	HWE	N	%	MAF	HWE	*P*
AA	401	65.3	C: 18.6%	0.110	409	63.6	C: 20.6%	0.398	0.080
AC	198	32.3			203	31.6			
CC	15	2.4			31	4.8			
NOS1									
**rs7977109**									
AA	226	36.9	G: 40.5%	0.154	227	35.3	G: 39.9%	0.411	0.246
AG	278	45.3			319	49.6			
GG	109	17.8			97	15.1			
ORMDL3									
**rs12603332**									
CC	176	29.2	T: 46.7%	0.462	200	31.3	T: 44.6%	0.523	0.587
CT	291	48.3			308	48.2			
TT	136	22.5			131	20.5			
**rs4065275**									
GG	174	29.7	A: 45.1%	0.739	200	32.5	A: 42.4%	0.509	0.372
GA	295	50.3			309	50.2			
AA	117	20.0			106	17.2			
TBXA2R									
**rs4523**									
TT	264	43.6	C: 33.7%	0.785	242	37.7	C: 39.5%	0.247	**0.008**
TC	275	45.4			292	45.6			
CC	67	11.0			107	16.7			
TNF-α									
**rs1799964**									
TT	228	37.4	C: 38.4%	0.609	297	46.3	C: 31.2%	0.270	**0.001**
TC	295	48.3			289	45.0			
CC	87	14.3			56	8.7			
**rs1800630**									
CC	424	69.4	A: 16.6%	0.885	449	69.8	A: 16.7%	0.395	0.746
CA	171	28.0			173	26.9			
AA	16	2.6			21	3.3			
**rs1800629**									
GG	570	93.1	A: 3.5%	0.534	560	87.1	A: 6.6%	1.000	**0.002**
GA	41	6.7			81	12.6			
AA	1	0.2			2	0.3			

MAF: minor allele frequency; HWE: Hardy-Weinberg Equilibrium. *P* values were calculated using Chi-square test.

Supplementary Table S2: Mean lung function parameters (FEV1 and FVC) and 95% CI for the 18 SNP genotypes for Inuit residing in Greenland and Denmark.

		Greenland			Denmark		
		N	Mean (95% CI)	*P*	N	Mean (95% CI)	*P*
***ADAM33* rs612709**							
FEV1 (L)	GG	538	3.15 (3.07-3.24)		527	3.08 (3.00-3.15)	
	GA	73	3.16 (2.94-3.38)	0.668	85	3.23 (3.02-3.43)	0.590
	AA	1	1.55 (-)	-	5	2.68 (1.69-3.67)	0.201
**Overall *P***				0.668			0.371
FVC (L)	GG	532	3.98 (3.88-4.08)		510	3.83 (3.74-3.92)	
	GA	73	3.93 (3.67-4.19)	0.333	86	3.96 (3.71-4.20)	0.077
	AA	1	2.40 (-)	-	5	3.44 (2.32-4.56)	0.291
**Overall *P***				0.333			0.236
**rs528557**							
FEV1 (L)	CC	491	3.15 (3.06-3.24)		447	3.05 (2.97-3.12)	
	CG	106	3.14 (2.96-3.33)	0.412	156	3.24 (3.09-3.39)	0.520
	GG	10	3.29 (2.66-3.92)	0.475	12	3.02 (2.59-3.44)	0.711
**Overall *P***				0.572			0.745
FVC (L)	CC	485	3.98 (3.87-4.08)		432	3.78 (3.69-3.88)	
	CG	106	3.92 (3.70-4.15)	0.127	155	4.00 (3.82-4.19)	0.258
	GG	10	4.08 (3.37-4.79)	0.260	12	3.80 (3.31-4.28)	0.453
**Overall *P***				0.183			0.370
**rs44707**							
FEV1 (L)	AA	275	3.23 (3.11-3.35)		276	3.05 (2.95-3.15)	
	AC	268	3.06 (2.94-3.18)	0.058	285	3.15 (3.04-3.26)	0.783
	CC	64	3.25 (3.04-3.46)	0.913	54	2.98 (2.76-3.20)	0.589
**Overall *P***				0.146			0.782
FVC (L)	AA	274	4.04 (3.89-4.18)		270	3.79 (3.67-3.91)	
	AC	263	3.88 (3.73-4.02)	0.105	277	3.90 (3.77-4.03)	0.956
	CC	64	4.10 (3.83-4.37)	0.806	52	3.73 (3.46-3.99)	0.576
**Overall *P***				0.199			0.850
**rs2787094**							
FEV1 (L)	GG	488	3.16 (3.07-3.24)		468	3.06 (2.98-3.13)	
	GC	117	3.16 (2.98-3.34)	0.787	139	3.24 (3.09-3.39)	**0.043**
	CC	5	2.74 (1.63-3.85)	0.217	10	2.83 (2.27-3.39)	0.189
**Overall *P***				0.456			**0.045**
FVC (L)	GG	482	3.98 (3.87-4.09)		454	3.81(3.71-3.91)	
	GC	117	3.98 (3.76-4.20)	0.650	138	3.96 (3.79-4.14)	0.249
	CC	5	3.53 (2.23-4.83)	0.243	9	3.52 (2.72-4.32)	**0.045**
**Overall *P***				0.466			0.058
***ALOX5* rs4986832**							
FEV1 (L)	GG	540	3.16 (3.08-3.25)		503	3.06 (2.98-3.13)	
	GA	65	3.09 (2.82-3.36)	0.391	106	3.26 (3.09-3.44)	0.730
	AA	3	2.60 (-)	0.095	8	3.16 (2.59-3.72)	0.206
**Overall *P***				0.177			0.412
FVC (L)	GG	535	3.99 (3.89-4.09)		491	3.80 (3.71-3.89)	
	GA	64	3.87 (3.55-4.18)	0.223	103	4.02 (3.80-4.24)	0.785
	AA	3	3.48 (-)	0.130	7	3.99 (3.13-4.85)	0.319
**Overall *P***				0.158			0.593
**rs892690**							
FEV1 (L)	GG	271	3.21 (3.09-3.32)		208	3.11(2.99-3.23)	

		Greenland			Denmark		
		N	Mean (95% CI)	*P*	N	Mean (95% CI)	*P*
	GA	276	3.07 (2.95-3.19)	0.311	297	3.07 (2.98-3.17)	0.905
	AA	56	3.30 (3.04-3.56)	0.965	105	3.10 (2.93-3.28)	0.997
**Overall *P***				0.561			0.991
FVC (L)	GG	270	4.06 (3.91-4.20)		201	3.89 (3.75-4.04)	
	GA	272	3.85 (3.71-4.00)	0.159	291	3.80 (3.67-3.92)	0.298
	AA	56	4.15 (3.83-4.47)	0.950	102	3.85 (3.65-4.06)	0.651
**Overall *P***				0.347			0.582
**rs2115819**							
FEV1 (L)	TT	170	3.24 (3.10-3.38)		111	3.06 (2.90-3.23)	
	CT	302	3.12 (3.00-3.23)	0.511	319	3.15 (3.05-3.24)	0.720
	CC	135	3.11 (2.94-3.28)	0.355	183	3.04 (2.91-3.16)	0.731
**Overall *P***				0.640			0.929
FVC (L)	TT	170	4.07 (3.90-4.25)		110	3.82 (3.62-4.02)	
	CT	298	3.92 (3.78-4.07)	0.566	309	3.90 (3.78-4.02)	0.477
	CC	133	3.94 (3.75-4.14)	0.412	178	3.78 (3.62-3.93)	0.647
**Overall *P***				0.705			0.776
***LT-α* rs2844484**							
FEV1 (L)	CC	439	3.16 (3.07-3.25)		361	2.99 (2.90-3.08)	
	CT	151	3.11(2.94-3.27)	0.952	228	3.22 (3.11-3.34)	**0.036**
	TT	15	3.38 (2.80-3.95)	0.247	27	3.36 (2.99-3.74)	0.581
**Overall *P***				0.510			0.109
FVC (L)	CC	435	3.98 (3.87-4.09)		353	3.72 (3.61-3.82)	
	CT	150	3.92 (3.71-4.12)	0.932	220	3.99 (3.85-4.13)	**0.041**
	TT	14	4.39 (3.68-5.10)	0.212	27	4.20 (3.73-4.68)	0.470
**Overall *P***				0.458			0.115
**rs909253**							
FEV1 (L)	TT	180	3.23 (3.07-3.39)		193	3.09 (2.97-3.22)	
	TC	315	3.18 (3.07-3.29)	0.641	318	3.14 (3.04-3.23)	0.086
	CC	112	2.94 (2.78-3.10)	0.492	105	2.95 (2.79-3.10)	0.970
**Overall *P***				0.513			0.158
FVC (L)	TT	178	4.13 (3.94-4.32)		189	3.83 (3.68-3.98)	
	TC	313	3.98 (3.85-4.11)	0.619	310	3.90 (3.78-4.02)	0.081
	CC	110	3.67 (3.47-3.87)	0.146	101	3.67 (3.48-3.85)	0.703
**Overall *P***				0.335			0.193
**rs1041981**							
FEV1 (L)	CC	183	3.22 (3.07-3.38)		194	3.10 (2.98-3.23)	
	CA	314	3.19 (3.08-3.30)	0.546	316	3.13 (3.04-3.23)	0.112
	AA	112	2.92 (2.76-3.08)	0.449	102	2.94 (2.78-3.10)	0.928
**Overall *P***				0.401			0.183
FVC (L)	CC	181	4.12 (3.93-4.31)		190	3.84 (3.69-3.99)	
	CA	312	3.99 (3.86-4.12)	0.735	308	3.90 (3.78-4.02)	0.095
	AA	110	3.65 (3.44-3.85)	0.134	98	3.66 (3.47-3.85)	0.755
**Overall *P***				0.292			0.217
***LTC4S* rs730012**							
FEV1 (L)	*AA*	399	3.12 (3.02-3.22)		391	3.09 (3.00-3.17)	
	AC	198	3.22 (3.09-3.35)	0.479	196	3.09 (2.97-3.20)	0.326
	CC	15	3.14 (2.68-3.60)	0.839	30	3.24 (2.92-3.56)	0.893
**Overall *P***				0.772			0.592
FVC (L)	*AA*	396	3.94 (3.82-4.06)		382	3.84 (3.73-3.95)	
	AC	195	4.04 (3.87-4.20)	0.608	190	3.82 (3.69-3.96)	0.259

		Greenland			Denmark		
		N	Mean (95% CI)	*P*	N	Mean (95% CI)	*P*
	CC	15	3.89 (3.39-4.39)	0.940	29	3.94 (3.52-4.34)	0.865
**Overall *P***				0.869			0.529
***NOS1* rs7977109**							
FEV1 (L)	AA	225	3.15 (3.02-3.28)		218	3.02 (2.91-3.13)	
	AG	278	3.19 (3.08-3.30)	0.967	306	3.14 (3.04-3.24)	0.523
	GG	108	3.07 (2.86-3.29)	0.303	93	3.12 (2.94-3.30)	0.870
**Overall *P***				0.534			0.810
FVC (L)	AA	224	3.96 (3.80-4.11)		212	3.77 (3.63-3.90)	
	AG	277	4.00 (3.87-4.14)	0.912	299	3.90 (3.77-4.02)	0.728
	GG	104	3.94 (3.67-4.20)	0.695	90	3.83 (3.62-4.05)	0.736
**Overall *P***				0.885			0.816
***ORMDL3* rs12603332**							
FEV1 (L)	CC	175	3.29 (3.14-3.43)		189	3.00 (2.87-3.12)	
	CT	290	3.07 (2.95-3.18)	**0.007**	297	3.12 (3.02-3.22)	0.241
	TT	136	3.17 (3.00-3.35)	0.180	127	3.18 (3.03-3.33)	0.374
**Overall *P***				**0.027**			0.470
FVC (L)	CC	175	4.13 (3.95-4.31)		188	3.73 (3.58-3.89)	
	CT	286	3.89 (3.75-4.03)	**0.010**	286	3.87 (3.75-3.99)	0.321
	TT	134	3.96 (3.75-4.17)	0.091	123	3.94 (3.76-4.12)	0.645
**Overall *P***				**0.033**			0.610
**rs4065275**							
FEV1 (L)	GG	173	3.29 (3.15-3.44)		189	2.98 (2.86-3.11)	
	GA	294	3.06 (2.95-3.17)	**0.006**	298	3.12 (3.02-3.22)	0.238
	AA	117	3.19 (3.01-3.38)	0.168	104	3.19 (3.03-3.36)	0.265
**Overall *P***				**0.023**			0.405
FVC (L)	GG	173	4.14 (3.96-4.32)		188	3.72 (3.57-3.87)	
	GA	290	3.88 (3.74-4.02)	**0.007**	287	3.87 (3.75-3.99)	0.293
	AA	115	3.98 (3.75-4.20)	0.080	101	3.94 (3.74-4.14)	0.469
**Overall *P***				**0.025**			0.553
***TBXA2R* rs4523**							
FEV1 (L)	TT	263	3.21 (3.10-3.33)		232	2.99 (2.87-3.10)	
	TC	275	3.05 (2.93-3.17)	0.081	284	3.17 (3.07-3.27)	0.413
	CC	66	3.37 (3.13-3.61)	0.999	99	3.13 (2.95-3.31)	0.254
**Overall *P***				0.183			0.486
FVC (L)	TT	263	4.05 (3.91-4.19)		226	3.75 (3.61-3.89)	
	TC	271	3.84 (3.69-3.99)	0.059	274	3.92 (3.79-4.04)	0.136
	CC	64	4.26 (3.97-4.55)	0.734	99	3.85 (3.65-4.05)	0.086
**Overall *P***				0.162			0.160
***TNF-α* rs1799964**							
FEV1 (L)	TT	227	3.00 (2.88-3.13)		286	3.13 (3.03-3.23)	
	CT	294	3.25 (3.13-3.37)	0.621	276	3.10 (2.99-3.21)	0.534
	CC	87	3.20 (2.99-3.42)	0.711	54	2.86 (2.65-3.07)	0.201
**Overall *P***				0.870			0.424
FVC (L)	TT	224	3.75 (3.60-3.90)		277	3.89 (3.77-4.01)	
	CT	291	4.11 (3.97-4.25)	0.955	269	3.84 (3.71-3.97)	0.225
	CC	87	4.09 (3.84-4.35)	0.613	54	3.55 (3.31-3.78)	0.135
**Overall *P***				0.868			0.232
**rs1800630**							
FEV1 (L)	CC	422	3.09 (3.00-3.19)		430	3.08 (3.00-3.16)	
	CA	171	3.29 (3.14-3.45)	0.705	167	3.12 (2.97-3.27)	0.589

		Greenland			Denmark		
		N	Mean (95% CI)	*P*	N	Mean (95% CI)	*P*
	AA	16	3.28 (2.63-3.93)	0.681	20	3.11 (2.71-3.50)	0.683
**Overall *P***				0.868			0.773
FVC (L)	CC	418	3.89 (3.78-4.00)		418	3.85 (3.75-3.94)	
	CA	169	4.17 (3.98-4.36)	0.463	163	3.82 (3.64-4.00)	0.100
	AA	16	4.24 (3.42-5.05)	0.232	20	3.90 (3.42-4.38)	0.432
**Overall *P***				0.406			0.161
**rs1800629**							
FEV1 (L)	GG	568	3.15 (3.07-3.24)		534	3.07 (3.00-3.14)	
	GA	41	3.05 (2.74-3.36)	0.414	81	3.25 (3.06-3.43)	0.888
	AA	1	2.90 (-)	-	2	3.30 (-)	-
**Overall *P***				0.414			0.888
FVC (L)	GG	563	3.98 (3.88-4.08)		523	3.81(3.72-3.90)	
	GA	40	3.80 (3.42-4.18)	0.160	76	4.04 (3.81-4.28)	0.518
	AA	1	3.45 (-)	-	2	4.23 (-)	-
**Overall *P***				0.160			0.518

CI: confidence interval. The associations (overall *P* value) were investigated using general linear model between SNPs and lung function, while *P* values in column were calculated for comparisons of heterozygotes and homozygotes vs reference allele respectively. All *P* values were adjusted by gender, age, height, ethnicity and smoking condition.

Supplementary Table S3: The prevalence and odds ratios of ever asthma in Inuit the 18 SNP genotypes in Greenlandic and Danish populations.

	Greenland Ever asthma					Denmark Ever asthma				
	**N**	**n**	**%**	**OR**	***P***	**N**	**n**	**%**	**OR**	***P***
***ADAM33* rs612709**										
GG	540	47	8.7	1.00		548	51	9.3	1.00	
GA	73	5	6.8	0.83	0.719	89	8	9.0	0.92	0.980
AA	1	0	0.0	-	-	5	0	0.0	0.00	0.999
**Overall *P***					0.719					1.000
**rs528557**										
CC	493	43	8.7	1.00		465	44	9.5	1.00	
CG	106	7	6.6	0.76	0.470	162	15	9.3	0.94	0.851
GG	10	1	10.0	1.27	0.749	12	0	0.0	-	-
**Overall *P***					0.721				0.00	0.999
**rs44707**										
AA	276	22	8.0	1.00		291	22	7.6	1.00	
AC	269	21	7.8	0.94	0.655	294	30	10.2	1.38	0.244
CC	64	8	12.5	2.18	0.085	55	6	10.9	1.52	0.428
**Overall *P***					0.124					0.466
**rs2787094**										
GG	490	41	8.4	1.00		488	43	8.8	1.00	
GC	108	9	7.7	0.98	0.893	144	16	11.1	1.26	0.305
CC	4	1	20.0	2.72	0.395	10	0	0.0	0.00	0.999
**Overall *P***					0.707					0.590
***ALOX5* rs4986832**										
GG	542	44	8.1	1.00		524	50	9.5	1.00	
GA	65	7	10.8	1.24	0.651	110	8	7.3	0.71	0.414
AA	3	1	33.3	5.38	0.234	8	1	12.5	1.63	0.579
**Overall *P***					0.454					0.600
**rs892690**										
GG	272	20	7.4	1.00		211	21	10.0	1.00	
GA	277	23	8.3	1.13	0.798	312	30	9.6	0.96	0.773
AA	56	9	16.1	2.78	**0.022**	112	8	7.1	0.68	0.338
**Overall *P***					0.058					0.628
**rs2115819**										
TT	170	13	7.6	1.00		116	11	9.5	1.00	
CT	304	26	8.6	1.07	0.832	331	29	8.8	0.93	0.832
CC	135	13	9.6	1.34	0.537	191	18	9.4	1.03	0.898
**Overall *P***					0.812					0.978
***LT-α* rs2844484**										
CC	440	37	8.4	1.00		380	39	10.3	1.00	
CT	152	13	8.6	0.86	0.731	234	18	7.7	0.72	0.267
TT	15	1	6.7	0.82	0.887	27	2	7.4	0.64	0.558
**Overall *P***					0.937					0.487
**rs909253**										
TT	180	16	8.9	1.00		199	19	9.5	1.00	
TC	317	24	7.6	0.99	0.890	332	26	7.8	0.86	0.645
CC	112	12	10.7	1.34	0.562	110	14	12.7	1.50	0.286
**Overall *P***					0.732					0.226
**rs1041981**										

	Greenland Ever asthma					Denmark Ever asthma				
	**N**	**n**	**%**	**OR**	***P***	**N**	**n**	**%**	**OR**	***P***
CC	183	16	8.7	1.00		200	19	9.5	1.00	
CA	316	23	7.3	0.97	0.858	330	26	7.9	0.88	0.572
AA	112	12	10.7	1.34	0.570	107	13	12.1	1.44	0.328
**Overall *P***					0.724					0.304
***LTC4S* rs730012**										
AA	401	40	10.0	1.00		409	39	9.5	1.00	
AC	198	11	5.6	0.58	0.162	203	19	9.4	0.96	0.942
CC	15	1	6.7	0.65	0.665	30	1	3.3	0.34	0.326
**Overall *P***					0.356					0.617
***NOS1* rs7977109**										
AA	226	25	11.1	1.00		227	23	10.1	1.00	
AG	278	17	6.1	0.56	0.086	318	23	7.2	0.68	0.190
GG	109	9	8.6	0.53	0.158	97	13	13.4	1.33	0.418
**Overall *P***					0.149					0.135
***ORMDL3* rs12603332**										
CC	176	16	9.1	1.00		199	18	9.0	1.00	
CT	291	22	7.6	0.64	0.337	308	27	8.8	0.98	0.960
TT	136	13	9.6	0.91	0.919	131	13	9.9	1.11	0.642
**Overall *P***					0.573					0.850
**rs4065275**										
GG	174	16	9.2	1.00		199	18	9.0	1.00	
GA	295	23	7.8	0.66	0.376	309	27	8.7	0.97	0.948
AA	117	9	7.7	0.76	0.628	106	10	9.4	1.02	0.848
**Overall *P***					0.673					0.967
***TBXA2R* rs4523**										
TT	264	22	8.3	1.00		242	21	8.7	1.00	
TC	275	25	9.1	0.91	0.843	291	26	8.9	0.97	0.948
CC	67	4	6.0	0.69	0.613	107	11	10.3	1.17	0.519
**Overall *P***					0.879					0.751
***TNF-α* rs1799964**										
TT	228	21	9.2	1.00		297	29	9.8	1.00	
TC	295	24	8.1	0.93	0.950	288	22	7.6	0.76	0.414
CC	87	7	8.0	0.80	0.641	56	8	14.3	1.40	0.456
**Overall *P***					0.871					0.414
**rs1800630**										
CC	424	35	8.3	1.00		449	45	10.0	1.00	
CA	171	14	8.2	0.97	0.876	172	11	6.4	0.59	0.168
AA	16	2	12.5	0.75	0.713	21	3	14.3	1.34	0.742
**Overall *P***					0.918					0.343
**rs1800629**										
GG	570	49	8.6	1.00		559	51	9.1	1.00	
GA	41	3	7.3	0.90	0.865	81	8	9.9	1.10	0.822
AA	1	0	0.0	-	-	2	0	0.0	-	-
**Overall *P***					0.865					0.822

OR: odds ratio. The associations (overall *P* value) were investigated using logistic regression analyses between SNPs and disease prevalence, while *P* values in column were calculated for comparisons of heterozygotes and homozygotes vs reference allele respectively. All *P* values and ORs were adjusted by gender, age, height, ethnicity and smoking condition.

Supplementary Table S4: The prevalence and odds ratios of bronchitis in Inuit for the 18 SNP genotypes in Greenlandic and Danish populations.

	Greenland Bronchitis					Denmark Bronchitis				
	**N**	**n**	**%**	**OR**	***P***	**N**	**n**	**%**	**OR**	***P***
***ADAM33* rs612709**										
GG	539	138	25.6	1.00		548	76	13.9	1.00	
GA	73	17	23.3	0.90	0.717	89	13	14.6	1.12	0.727
AA	1	1	100.0	-	-	5	0	0.0	-	-
**Overall *P***					0.717					0.727
**rs528557**										
CC	492	127	25.8	1.00		464	65	14.0	1.00	
CG	106	27	25.5	0.90	0.930	163	22	13.5	1.02	0.865
GG	10	1	10.0	0.29	0.250	12	1	8.3	0.57	0.613
**Overall *P***					0.564					0.862
**rs44707**										
AA	276	64	23.2	1.00		290	38	13.1	1.00	
AC	268	76	28.4	0.21	0.149	295	40	13.6	1.10	0.813
CC	64	16	25.0	0.66	0.644	55	11	20.0	1.77	0.145
**Overall *P***					0.450					0.334
**rs2787094**										
GG	489	124	25.4	1.00		488	71	14.5	1.00	
GC	117	29	24.8	0.84	0.801	144	17	11.8	0.83	0.488
CC	5	3	60.0	0.14	0.118	10	1	10.0	0.66	0.656
**Overall *P***					0.319					0.722
***ALOX5* rs4986832**										
GG	541	137	25.3	1.00		523	73	14.0	1.00	
GA	65	17	26.2	0.83	0.680	111	14	12.6	0.93	0.955
AA	3	1	33.3	0.92	0.865	8	2	25.0	2.01	0.435
**Overall *P***					0.974					0.733
**rs892690**										
GG	271	71	26.2	1.00		211	27	12.8	1.00	
GA	277	69	24.9	0.78	0.809	312	47	15.1	1.17	0.515
AA	56	16	28.6	0.58	0.541	112	15	13.4	1.07	0.862
**Overall *P***					0.771					0.800
**rs2115819**										
TT	169	41	24.3	1.00		116	16	13.8	1.00	
CT	304	81	26.6	0.56	0.547	331	41	12.4	0.88	0.724
CC	135	34	25.2	0.82	0.849	191	31	16.2	1.17	0.641
**Overall *P***					0.840					0.587
***LT-α* rs2844484**										
CC	440	113	25.7	1.00		379	63	16.6	1.00	
CT	151	36	23.8	0.90	0.647	235	18	7.7	0.41	**0.003**
TT	15	5	33.3	1.54	0.419	27	7	25.9	1.92	0.171
**Overall *P***					0.624					**0.002**
**rs909253**										
TT	180	45	25.0	1.00		200	24	12.0	1.00	
TC	316	79	25.0	1.06	0.720	331	42	12.7	1.02	0.945
CC	112	32	28.6	1.33	0.318	110	23	20.9	1.91	0.060
**Overall *P***					0.597					0.090
**rs1041981**										

	Greenland Bronchitis					Denmark Bronchitis				
	**N**	**n**	**%**	**OR**	***P***	**N**	**n**	**%**	**OR**	***P***
CC	183	46	25.1	1.00		201	24	11.9	1.00	
CA	315	78	24.8	0.98	0.778	329	42	12.8	1.08	0.924
AA	112	32	28.6	1.19	0.339	107	23	21.5	2.02	**0.047**
**Overall *P***					0.615					0.070
***LTC4S* rs730012**										
AA	400	108	27.0	1.00		408	52	12.7	1.00	
AC	198	43	21.7	0.78	0.306	203	33	16.3	1.34	0.238
CC	15	5	33.3	1.36	0.572	31	4	12.9	0.99	0.905
**Overall *P***					0.470					0.497
***NOS1* rs7977109**										
AA	225	53	23.6	1.00		226	33	14.6	1.00	
AG	278	73	26.3	1.17	0.418	319	41	12.9	0.87	0.503
GG	109	30	27.5	1.17	0.566	97	15	15.5	1.14	0.791
**Overall *P***					0.700					0.666
***ORMDL3* rs12603332**										
CC	176	43	24.4	1.00		199	23	11.6	1.00	
CT	290	73	25.2	1.01	0.786	308	49	15.9	1.51	0.118
TT	136	39	28.7	1.20	0.426	131	17	13.0	1.13	0.768
**Overall *P***					0.717					0.245
**rs4065275**										
GG	174	43	24.7	1.00		199	23	11.6	1.00	
GA	294	74	25.2	1.00	0.847	309	49	15.9	1.50	0.118
AA	117	33	28.2	1.19	0.458	106	14	13.2	1.15	0.766
**Overall *P***					0.740					0.255
***TBXA2R* rs4523**										
TT	264	71	26.9	1.00		241	36	14.9	1.00	
TC	274	70	25.5	0.96	0.848	292	34	11.6	0.86	0.563
CC	67	14	20.9	0.72	0.343	107	19	17.8	1.39	0.229
**Overall *P***					0.636					0.232
***TNF-α* rs1799964**										
TT	227	64	28.2	1.00		297	44	14.8	1.00	
TC	295	68	23.1	0.70	0.117	288	36	12.5	0.80	0.356
CC	87	24	27.6	0.88	0.611	56	9	16.1	1.11	0.787
**Overall *P***					0.291					0.561
**rs1800630**										
CC	423	110	26.0	1.00		449	62	13.8	1.00	
CA	171	38	22.2	0.78	0.295	172	24	14.0	1.02	0.965
AA	16	8	50.0	3.22	**0.031**	21	3	14.3	1.10	0.852
**Overall *P***					**0.042**					0.983
**rs1800629**										
GG	569	139	24.4	1.00		559	80	14.3	1.00	
GA	41	17	41.5	2.41	**0.010**	81	9	11.1	0.82	0.595
AA	1	0	0.0	-	-	2	0	0.0	-	-
**Overall *P***					**0.010**					0.595

OR: odds ratio. The associations (overall *P* value) were investigated using logistic regression analyses between SNPs and disease prevalence, while *P* values in column were calculated for comparisons of heterozygotes and homozygotes vs reference allele respectively. All *P* values and ORs were adjusted by gender, age, height, ethnicity and smoking condition.

Supplementary Table S5: The prevalence and odds ratios of rhinitis in Inuit for the 18 SNP genotypes in Greenlandic and Danish populations.

	Greenland Rhinitis					Denmark Rhinitis				
	**N**	**n**	**%**	**OR**	***P***	**N**	**n**	**%**	**OR**	***P***
***ADAM33* rs612709**										
GG	357	150	42.0	1.00		544	325	59.7	1.00	
GA	46	22	47.8	1.38	0.318	88	56	63.6	1.18	0.503
AA	1	0	0.0	-	-	5	5	100.0	-	-
**Overall *P***					0.318					0.503
**rs528557**										
CC	323	136	42.1	1.00		461	279	60.5	1.00	
CG	73	33	45.2	1.15	0.576	161	98	60.9	1.02	0.686
GG	4	1	25.0	0.45	0.577	12	8	66.7	1.31	0.664
**Overall *P***					0.721					0.849
**rs44707**										
AA	184	76	41.3	1.00		286	168	58.7	1.00	
AC	169	75	44.4	1.18	0.516	294	179	60.9	1.07	0.762
CC	47	19	40.4	1.04	0.927	55	37	67.3	1.33	0.387
**Overall *P***					0.804					0.686
**rs2787094**										
GG	322	135	41.9	1.00		484	287	59.3	1.00	
GC	77	33	42.9	1.03	0.906	143	91	63.6	1.13	0.397
CC	4	3	75.0	4.21	0.226	10	8	80.0	3.13	0.150
**Overall *P***					0.479					0.262
***ALOX5* rs4986832**										
GG	347	154	44.4	1.00		519	314	60.5	1.00	
GA	51	15	29.4	0.53	**0.049**	110	66	60.0	0.97	0.975
AA	2	1	50.0	1.19	0.916	8	6	75.0	2.36	0.298
**Overall *P***					0.142					0.579
**rs892690**										
GG	177	66	37.3	1.00		209	125	59.8	1.00	
GA	183	85	46.4	1.47	0.078	310	195	62.9	1.17	0.452
AA	39	19	48.7	1.61	0.179	111	62	55.9	0.85	0.499
**Overall *P***					0.150					0.393
**rs2115819**										
TT	116	44	37.9	1.00		114	71	62.3	1.00	
CT	195	90	46.2	1.37	0.182	331	200	60.4	0.94	0.699
CC	90	38	42.2	1.19	0.585	188	113	60.1	0.99	0.896
**Overall *P***					0.402					0.912
***LT-α* rs2844484**										
CC	284	123	43.3	1.00		377	229	60.7	1.00	
CT	103	43	41.7	0.92	0.767	232	139	59.9	0.92	0.697
TT	12	5	41.7	0.95	0.942	27	17	63.0	1.04	0.868
**Overall *P***					0.956					0.902
**rs909253**										
TT	119	54	45.5	1.00		200	120	60.0	1.00	
TC	206	83	40.3	0.83	0.382	327	199	60.9	1.04	0.861
CC	75	34	45.3	1.11	0.852	109	66	60.6	1.03	0.999
**Overall *P***					0.528					0.980
**rs1041981**										

	Greenland Rhinitis					Denmark Rhinitis				
	**N**	**n**	**%**	**OR**	***P***	**N**	**n**	**%**	**OR**	***P***
CC	121	55	45.5	1.00		201	121	60.2	1.00	
CA	207	83	40.1	0.82	0.344	325	197	60.6	1.02	0.925
AA	74	34	45.9	1.12	0.817	106	64	60.4	1.02	0.978
**Overall *P***					0.463					0.992
***LTC4S* rs730012**										
AA	265	117	44.2	1.00		406	252	62.1	1.00	
AC	130	53	40.8	0.89	0.707	200	114	57.0	0.86	0.429
CC	9	2	22.2	0.41	0.292	31	20	64.5	1.20	0.439
**Overall *P***					0.552					0.486
***NOS1* rs7977109**										
AA	146	66	45.2	1.00		224	138	61.6	1.00	
AG	189	75	39.7	0.79	0.283	316	188	59.5	0.87	0.381
GG	68	30	44.1	0.92	0.840	97	60	61.9	0.96	0.814
**Overall *P***					0.543					0.673
***ORMDL3* rs12603332**										
CC	109	49	45.0	1.00		199	123	61.8	1.00	
CT	193	79	40.9	0.88	0.683	304	172	56.6	0.79	0.303
TT	94	41	43.6	0.95	0.830	130	88	67.7	1.22	0.384
**Overall *P***					0.920					0.178
**rs4065275**										
GG	107	48	44.9	1.00		199	123	61.8	1.00	
GA	196	81	41.3	0.91	0.949	305	172	56.4	0.78	0.289
AA	83	36	43.4	0.96	0.968	105	74	70.5	1.36	0.210
**Overall *P***					0.978					0.091
***TBXA2R* rs4523**										
TT	173	78	45.1	1.00		240	146	60.8	1.00	
TC	178	77	43.3	1.01	0.917	289	173	59.9	0.89	0.443
CC	48	15	31.2	0.56	0.130	106	66	62.3	1.01	0.907
**Overall *P***					0.261					0.665
***TNF-α* rs1799964**										
TT	153	68	44.4	1.00		293	181	61.8	1.00	
TC	195	75	38.5	0.73	0.254	287	170	59.2	0.94	0.654
CC	52	25	53.8	1.35	0.327	56	34	60.7	0.94	0.928
**Overall *P***					0.153					0.904
**rs1800630**										
CC	287	127	44.3	1.00		445	266	59.8	1.00	
CA	105	42	40.0	0.80	0.376	171	105	61.4	1.12	0.554
AA	10	2	20.0	0.29	0.122	21	15	71.4	1.66	0.332
**Overall *P***					0.226					0.554
**rs1800629**										
GG	370	160	43.2	1.00		555	335	60.4	1.00	
GA	31	11	35.5	0.75	0.455	80	49	61.2	0.96	0.873
AA	1	1	100.0	-	-	2	2	100.0	-	-
**Overall *P***					0.455					0.873

OR: odds ratio. The associations (overall *P* value) were investigated using logistic regression analyses between SNPs and disease prevalence, while *P* values in column were calculated for comparisons of heterozygotes and homozygotes vs reference allele respectively. All *P* values and ORs were adjusted by gender, age, height, ethnicity and smoking condition.

Supplementary Table S6: The prevalence and odds ratios of dermatitis in Inuit the 18 SNP genotypes in Greenlandic and Danish populations.

	Greenland Dermatitis					Denmark Dermatitis				
	**N**	**n**	**%**	**OR**	***P***	**N**	**n**	**%**	**OR**	***P***
ADAM33 **rs612709**										
GG	540	318	58.9	1.00		548	200	36.5	1.00	
GA	73	35	47.9	0.62	0.063	89	31	34.8	0.92	0.773
AA	1	1	100.0	-	-	5	2	40.0	1.17	0.843
**Overall *P***					0.063					0.939
**rs528557**										
CC	493	286	58.0	1.00		464	166	35.8	1.00	
CG	106	60	56.6	0.96	0.831	163	60	36.8	1.06	0.911
GG	10	5	50.0	0.75	0.603	12	6	50.0	1.74	0.315
**Overall *P***					0.860					0.603
**rs44707**										
AA	276	150	54.3	1.00		290	99	34.1	1.00	
AC	269	163	60.6	1.35	0.077	295	114	38.6	1.20	0.201
CC	64	38	59.4	1.17	0.548	55	18	32.7	0.87	0.807
**Overall *P***					0.209					0.360
**rs2787094**										
GG	490	288	58.8	1.00		488	175	35.9	1.00	
GC	117	62	53.0	0.80	0.310	144	56	38.9	1.07	0.590
CC	5	3	60.0	1.11	0.939	10	2	20.0	0.45	0.328
**Overall *P***					0.593					0.519
ALOX5 **rs4986832**										
GG	542	310	57.2	1.00		523	196	37.5	1.00	
GA	65	39	60.0	1.30	0.606	111	35	31.5	0.76	0.282
AA	3	2	66.7	1.70	0.680	8	2	25.0	0.59	0.532
**Overall *P***					0.808					0.475
**rs892690**										
GG	272	160	58.8	1.00		211	80	37.9	1.00	
GA	277	159	57.4	0.92	0.696	312	112	35.9	0.94	0.782
AA	56	31	55.4	0.83	0.515	112	40	35.7	0.93	0.892
**Overall *P***					0.792					0.962
**rs2115819**										
TT	170	98	57.6	1.00		116	46	39.7	1.00	
CT	304	176	57.9	1.02	0.749	331	123	37.2	0.95	0.904
CC	135	76	56.3	0.97	0.902	191	63	33.0	0.77	0.394
**Overall *P***					0.894					0.578
LT-α **rs2844484**										
CC	440	240	54.5	1.00		379	148	39.1	1.00	
CT	152	100	65.8	1.63	**0.012**	235	77	32.8	0.74	0.124
TT	15	10	66.7	1.62	0.366	27	8	29.6	0.64	0.368
**Overall *P***					**0.035**					0.245
**rs909253**										
TT	180	112	62.2	1.00		200	65	32.5	1.00	
TC	317	183	57.7	0.82	0.343	331	130	39.3	1.39	0.077
CC	112	56	50.0	0.63	0.078	110	37	33.6	1.10	0.772
**Overall *P***					0.211					0.170
**rs1041981**										

	Greenland Dermatitis					Denmark Dermatitis				
	N	n	%	OR	*P*	N	n	%	OR	*P*
CC	183	114	62.3	1.00		201	66	32.8	1.00	
CA	316	184	58.2	0.83	0.388	329	130	39.5	1.39	0.080
AA	112	55	49.1	0.61	0.059	107	35	32.7	1.04	0.953
**Overall *P***					0.167					0.147
***LTC4S* rs730012**										
AA	401	224	55.9	1.00		408	158	38.7	1.00	
AC	198	125	63.1	1.33	0.110	203	68	33.5	0.80	0.163
CC	15	5	33.3	0.40	0.097	31	7	22.6	0.46	0.127
**Overall *P***					0.051					0.150
***NOS1* rs7977109**										
AA	226	123	54.4	1.00		226	80	35.4	1.00	
AG	278	162	58.3	1.11	0.581	319	117	36.7	1.06	0.659
GG	109	68	62.4	1.35	0.222	97	36	37.1	1.03	0.787
**Overall *P***					0.473					0.904
***ORMDL3* rs12603332**										
CC	176	102	58.0	1.00		199	65	32.7	1.00	
CT	291	168	57.7	1.03	0.986	308	118	38.3	1.30	0.135
TT	136	78	57.4	0.97	0.841	131	49	37.4	1.27	0.271
**Overall *P***					0.970					0.302
**rs4065275**										
GG	174	101	58.0	1.00		199	64	32.2	1.00	
GA	295	171	58.0	1.04	0.961	309	118	38.2	1.32	0.110
AA	117	66	56.4	0.94	0.730	106	39	36.8	1.25	0.308
**Overall *P***					0.911					0.268
***TBXA2R* rs4523**										
TT	264	158	59.8	1.00		241	88	36.5	1.00	
TC	275	150	54.5	0.85	0.296	292	107	36.6	1.02	0.928
CC	67	43	64.2	1.23	0.610	107	36	33.6	0.91	0.775
**Overall *P***					0.392					0.935
***TNF-α* rs1799964**										
TT	228	131	57.5	1.00		297	101	34.0	1.00	
TC	295	169	57.3	0.95	0.594	288	111	38.5	1.20	0.285
CC	87	50	57.5	0.97	0.778	56	20	35.7	1.03	0.990
**Overall *P***					0.866					0.534
**rs1800630**										
CC	424	244	57.5	1.00		449	158	35.2	1.00	
CA	171	98	57.3	0.96	0.676	172	69	40.1	1.24	0.294
AA	16	10	62.5	1.12	0.920	21	6	28.6	0.72	0.517
**Overall *P***					0.907					0.428
**rs1800629**										
GG	570	329	57.7	1.00		559	203	36.3	1.00	
GA	41	24	58.5	1.04	0.901	81	30	37.0	1.07	0.798
AA	1	1	100.0	-	-	2	0	0.0	-	-
**Overall *P***					0.901					0.798

OR: odds ratio. The associations (overall *P* value) were investigated using logistic regression analyses between SNPs and disease prevalence, while *P* values in column were calculated for comparisons of heterozygotes and homozygotes vs reference allele respectively. All *P* values and ORs were adjusted by gender, age, height, ethnicity and smoking condition.

Supplementary Table S7: The prevalence and odds ratios of atopy in Inuit the 18 SNP genotypes in Greenlandic and Danish populations.

	Greenland Atopy					Denmark Atopy				
	**N**	**n**	*%*	**OR**	***P***	**N**	**n**	*%*	**OR**	***P***
***ADAM33* rs612709**										
GG	110	26	23.6	1.00		440	108	24.5	1.00	
GA	15	4	26.7	0.92	0.897	71	12	16.9	0.55	0.116
AA	0	0	0.0	-	-	5	1	20.0	0.91	0.961
**Overall *P***					0.897					0.291
**rs528557**										
CC	107	25	23.4	1.00		375	92	24.5	1.00	
CG	17	5	29.4	1.20	0.757	129	27	20.9	0.74	0.259
GG	1	0	0.0	-	-	10	2	20.0	0.82	0.765
**Overall *P***					0.757					0.516
**rs44707**										
AA	57	13	22.8	1.00		241	53	22.0	1.00	
AC	51	11	21.6	0.89	0.795	236	56	23.7	1.03	0.812
CC	17	6	35.3	1.73	0.322	39	12	30.8	1.61	0.261
**Overall *P***					0.496					0.530
**rs2787094**										
GG	102	24	23.5	1.00		389	87	22.4	1.00	
GC	23	6	26.1	1.03	0.923	117	32	27.4	1.19	0.414
CC	0	0	0.0	-	-	10	2	20.0	1.01	0.936
**Overall *P***					0.923					0.716
***ALOX5* rs4986832**										
GG	115	28	24.3	1.00		421	97	23.0	1.00	
GA	10	2	20.0	0.78	0.605	89	20	22.5	0.97	0.801
AA	0	0	0.0	-	-	6	4	66.7	6.68	**0.029**
**Overall *P***					0.605					0.087
**rs892690**										
GG	47	10	21.3	1.00		171	46	26.9	1.00	
GA	62	14	22.6	1.09	0.618	247	57	23.1	0.87	0.489
AA	14	5	35.7	2.18	0.197	91	17	18.7	0.66	0.199
**Overall *P***					0.435					0.432
**rs2115819**										
TT	36	10	27.8	1.00		90	22	24.4	1.00	
CT	61	12	19.7	0.67	0.514	265	65	24.5	1.02	0.963
CC	28	8	28.6	1.14	0.844	157	34	21.7	0.90	0.719
**Overall *P***					0.672					0.869
***LT-α* rs2844484**										
CC	93	21	22.6	1.00		310	75	24.2	1.00	
CT	29	7	24.1	1.01	0.959	183	44	24.0	0.90	0.482
TT	3	2	66.7	5.70	0.111	22	2	9.1	0.27	0.082
**Overall *P***					0.869					0.195
**rs909253**										
TT	37	10	27.0	1.00		165	39	23.6	1.00	
TC	68	15	22.1	0.70	0.431	265	59	22.3	0.91	0.605
CC	20	5	25.0	0.76	0.694	86	23	26.7	1.29	0.426
**Overall *P***					0.734					0.440
**rs1041981**										

	Greenland Atopy					Denmark Atopy				
	N	n	%	OR	*P*	N	n	%	OR	*P*
CC	37	10	27.0	1.00		165	39	23.6	1.00	
CA	68	15	22.1	0.70	0.438	263	59	22.4	0.92	0.637
AA	19	5	26.3	0.83	0.801	85	22	25.9	1.23	0.528
**Overall *P***					0.731					0.566
***LTC4S* rs730012**										
AA	79	23	29.1	1.00		330	78	23.6	1.00	
AC	43	5	11.6	0.32	**0.022**	160	38	23.8	1.01	0.976
CC	3	2	66.7	4.87	0.473	26	5	19.2	0.77	0.545
**Overall *P***					**0.046**					0.831
***NOS1* rs7977109**										
AA	43	12	27.9	1.00		175	49	28.0	1.00	
AG	62	12	19.4	0.67	0.426	260	53	20.4	0.63	0.065
GG	20	6	30.0	1.24	0.878	81	19	23.5	0.75	0.386
**Overall *P***					0.636					0.180
***ORMDL3* rs12603332**										
CC	38	12	31.6	1.00		162	36	22.2	1.00	
CT	60	12	20.0	0.47	0.179	240	59	24.6	1.12	0.650
TT	26	6	23.1	0.57	0.440	111	26	23.4	1.08	0.732
**Overall *P***					0.180					0.895
**rs4065275**										
GG	38	12	31.6	1		163	36	22.1	1	
GA	60	12	20	0.47	0.172	241	59	24.5	1.13	0.623
AA	23	6	26.1	0.68	0.597	90	21	23.3	1.07	0.802
**Overall *P***					0.392					0.886
***TBXA2R* rs4523**										
TT	55	13	23.6	1		190	46	24.2	1	
TC	53	16	30.2	1.39	0.587	239	60	25.1	0.93	0.648
CC	17	1	5.9	0.2	0.132	86	15	17.4	0.64	0.233
**Overall *P***					0.216					0.490
***TNF-α* rs1799964**										
TT	41	11	26.8	1		228	57	25	1	
TC	64	16	25	1.05	0.833	241	51	21.2	0.84	0.451
CC	19	3	15.8	0.63	0.477	47	13	27.7	1.22	0.460
**Overall *P***					0.772					0.455
**rs1800630**										
CC	85	20	23.5	1		353	85	24.1	1	
CA	38	10	26.3	1.4	0.521	145	30	20.7	0.79	0.449
AA	2	0	0	-	-	18	6	33.3	1.54	0.390
**Overall *P***					0.814					0.471
**rs1800629**										
GG	115	28	24.3	1		448	103	23	1	
GA	10	2	20	0.85	0.851	66	18	27.3	1.15	0.650
AA	0	0	0	-	-	2	0	0	-	-
**Overall *P***					0.851					0.650

OR: odds ratio. The associations (overall *P* value) were investigated using logistic regression analyses between SNPs and disease prevalence, while *P* values in column were calculated for comparisons of heterozygotes and homozygotes vs reference allele respectively. All *P* values and ORs were adjusted by gender, age, height, ethnicity and smoking condition.

Supplementary Table S8A: The genotype - phenotype associations in the sensitivity analysis for Inuit population residing in Greenland.

SNP	FEV1	FVC	Ever asthma	Bronchitis	Rhinitis	Dermatitis	Atopy
ADAM33							
rs612709	0.717	0.253	1.000	0.987	0.650	0.258	0.959
rs528557	0.490	0.206	0.698	0.566	0.585	0.973	0.996
rs44707	0.205	0.217	0.082	0.299	0.794	0.175	0.418
rs2787094	0.357	0.311	0.704	0.325	0.500	0.618	0.825
ALOX5							
rs4986832	0.177	0.133	0.499	0.992	0.226	0.880	0.919
rs892690	0.313	0.166	**0.029**	0.846	0.213	0.849	0.247
rs2115819	0.510	0.572	0.625	0.900	0.606	0.974	0.815
LT-α							
rs2844484	0.584	0.493	0.998	0.528	0.911	**0.025**	0.731
rs909253	0.822	0.864	0.830	0.557	0.255	0.296	0.858
rs1041981	0.712	0.846	0.833	0.573	0.214	0.242	0.772
LTC4S							
rs730012	0.787	0.841	0.274	0.501	0.629	0.131	0.051
NOS1							
rs7977109	0.605	0.919	0.173	0.810	0.385	0.542	0.496
ORMDL3							
rs12603332	**0.014**	**0.009**	0.381	0.810	0.967	0.869	0.567
rs4065275	**0.011**	**0.005**	0.338	0.703	0.975	0.841	0.673
TBXA2R							
rs4523	0.175	0.178	0.621	0.656	0.280	0.566	0.221
TNF-α							
rs1799964	0.702	0.750	0.971	0.222	0.326	0.686	0.675
rs1800630	0.953	0.549	0.784	0.063	0.171	0.812	0.900
rs1800629	0.428	0.169	0.729	**0.023**	0.793	0.454	0.694

The associations (overall *P* value) were investigated using general linear model between SNPs and lung function or using logistic regression analyses between SNPs and disease prevalence. Number represents overall *P* value adjusted by gender, age, height, and smoking condition.

Supplementary Table S8B: The genotype - phenotype associations in the sensitivity analysis for Inuit population residing in Denmark.

SNP	FEV1	FVC	Ever asthma	Bronchitis	Rhinitis	Dermatitis	Atopy
ADAM33							
rs612709	0.911	0.856	0.778	0.997	0.802	0.794	0.807
rs528557	0.637	0.240	0.887	0.822	0.627	0.879	0.546
rs44707	0.208	0.102	0.279	0.591	0.843	0.661	0.316
rs2787094	0.473	0.090	0.593	0.982	0.291	0.954	0.417
ALOX5							
rs4986832	0.786	0.701	0.665	0.863	0.992	0.705	0.234
rs892690	0.554	0.273	0.821	0.752	0.298	0.404	0.365
rs2115819	0.945	0.987	0.600	0.460	0.574	0.335	0.799
LT-α							
rs2844484	0.165	0.093	0.868	**0.044**	0.959	0.911	0.554
rs909253	0.142	0.159	0.375	**0.019**	0.471	0.613	0.328
rs1041981	0.158	0.169	0.531	**0.014**	0.404	0.565	0.444
LTC4S							
rs730012	0.599	0.244	0.976	0.551	0.281	0.868	0.690
NOS1							
rs7977109	0.705	0.913	0.455	0.895	0.755	0.693	0.594
ORMDL3							
rs12603332	0.737	0.624	0.890	0.750	0.090	0.159	0.715
rs4065275	0.741	0.544	0.704	0.737	0.164	0.185	0.519
TBXA2R							
rs4523	0.282	0.230	0.668	0.109	0.159	0.569	0.560
TNF-α							
rs1799964	0.464	0.404	0.222	0.609	0.721	0.630	0.341
rs1800630	0.866	0.363	0.419	0.969	0.284	0.458	0.521
rs1800629	0.926	0.562	0.300	0.344	0.948	0.620	0.719

The associations (overall *P* value) were investigated using general linear model between SNPs and lung function or using logistic regression analyses between SNPs and disease prevalence. Number represents overall *P* value adjusted by gender, age, height, and smoking condition.

Supplementary Table S9: The significance (*P* values) of the interaction of place with genotype-phenotype associations

SNP	FEV1	FVC	Ever asthma	Bronchitis	Rhinitis	Dermatitis	Atopy
ADAM33							
rs612709	0.418	0.557	0.968	0.867	0.974	0.561	0.312
rs528557	0.775	0.430	0.999	0.917	0.717	0.611	0.653
rs44707	0.351	0.386	0.483	0.430	0.665	0.825	0.867
rs2787094	0.354	0.601	0.634	0.379	0.858	0.446	0.983
ALOX5							
rs4986832	0.579	0.657	0.523	0.944	0.188	0.377	0.850
rs892690	0.715	0.833	0.060	0.688	0.370	0.914	0.263
rs2115819	0.873	0.890	0.848	0.587	0.346	0.892	0.538
LT-α							
rs2844484	0.261	0.264	0.921	0.108	0.966	**0.009**	0.073
rs909253	0.594	0.152	0.864	0.568	0.547	0.100	0.795
rs1041981	0.666	0.180	0.931	0.520	0.527	0.129	0.844
LTC4S							
rs730012	0.379	0.398	0.489	0.256	0.346	0.081	0.054
NOS1							
rs7977109	0.753	0.999	0.307	0.566	0.892	0.776	0.793
ORMDL3							
rs12603332	**0.024**	**0.042**	0.766	0.389	0.571	0.577	0.426
rs4065275	**0.021**	**0.030**	0.781	0.400	0.359	0.539	0.415
TBXA2R							
rs4523	0.327	0.400	0.719	0.198	0.267	0.439	0.301
TNF-α							
rs1799964	0.685	0.205	0.518	0.901	0.474	0.697	0.505
rs1800630	0.693	0.159	0.462	0.335	0.190	0.563	0.800
rs1800629	0.710	0.334	0.936	0.115	0.799	1.000	0.597

Number represents *P* value.

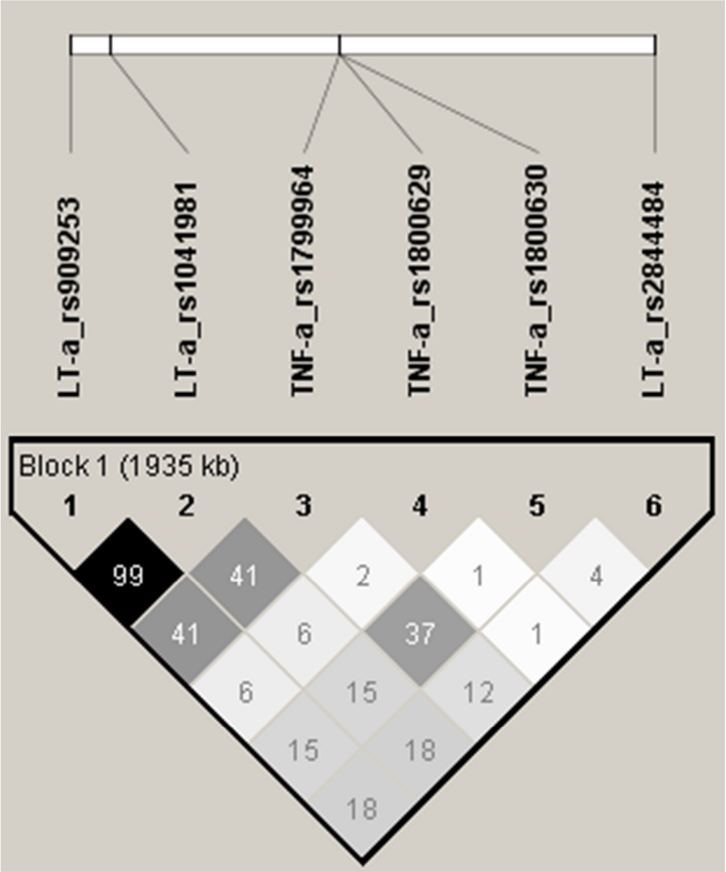

Supplementary Fig 1 - Example of the linkage disequilibrium (LD) in the selected single nucleotide polymorphisms (SNPs): *LT-α* and *TNF-α* SNPs are located in Chromosome 6. Haploview version 4.2 is utilized to visualise the LD. Black colour represents the strong correlation between the two SNPs.
